# Transcriptome Analysis in a Mouse Model of Premature Aging of Dentate Gyrus: Rescue of Alpha-Synuclein Deficit by Virus-Driven Expression or by Running Restores the Defective Neurogenesis

**DOI:** 10.3389/fcell.2021.696684

**Published:** 2021-08-17

**Authors:** Laura Micheli, Teresa Maria Creanza, Manuela Ceccarelli, Giorgio D’Andrea, Giacomo Giacovazzo, Nicola Ancona, Roberto Coccurello, Raffaella Scardigli, Felice Tirone

**Affiliations:** ^1^Institute of Biochemistry and Cell Biology, National Research Council, Rome, Italy; ^2^Institute of Intelligent Industrial Technologies and Systems for Advanced Manufacturing, National Research Council, Bari, Italy; ^3^Preclinical Neuroscience, European Center for Brain Research (CERC)/IRCCS Santa Lucia Foundation, Rome, Italy; ^4^Institute for Complex Systems, National Research Council, Rome, Italy; ^5^Institute of Translational Pharmacology, National Research Council, Rome, Italy

**Keywords:** adult neurogenesis, aging, dentate gyrus, neural stem cells, self-renewal, *alpha-synuclein* (*Snca*), physical exercise (running), RNA-seq

## Abstract

The dentate gyrus of the hippocampus and the subventricular zone are neurogenic niches where neural stem and progenitor cells replicate throughout life to generate new neurons. The *Btg1* gene maintains the stem cells of the neurogenic niches in quiescence. The deletion of *Btg1* leads to an early transient increase of stem/progenitor cells division, followed, however, by a decrease during adulthood of their proliferative capability, accompanied by apoptosis. Since a physiological decrease of neurogenesis occurs during aging, the *Btg1* knockout mouse may represent a model of neural aging. We have previously observed that the defective neurogenesis of the *Btg1* knockout model is rescued by the powerful neurogenic stimulus of physical exercise (running). To identify genes responsible for stem and progenitor cells maintenance, we sought here to find genes underlying this premature neural aging, and whose deregulated expression could be rescued by running. Through RNA sequencing we analyzed the transcriptomic profiles of the dentate gyrus isolated from *Btg1* wild-type or *Btg1* knockout adult (2-month-old) mice submitted to physical exercise or sedentary. In *Btg1* knockout mice, 545 genes were deregulated, relative to wild-type, while 2081 genes were deregulated by running. We identified 42 genes whose expression was not only down-regulated in the dentate gyrus of *Btg1* knockout, but was also counter-regulated to control levels by running in *Btg1* knockout mice, vs. sedentary. Among these 42 counter-regulated genes, *alpha-synuclein* (*Snca*), *Fos*, *Arc* and *Npas4* showed significantly greater differential regulation. These genes control neural proliferation, apoptosis, plasticity and memory and are involved in aging. In particular, Snca expression decreases during aging. We tested, therefore, whether an Snca-expressing lentivirus, by rescuing the defective Snca levels in the dentate gyrus of *Btg1* knockout mice, could also reverse the aging phenotype, in particular the defective neurogenesis. We found that the exogenous expression of Snca reversed the *Btg1* knockout-dependent decrease of stem cell proliferation as well as the increase of progenitor cell apoptosis. This indicates that Snca has a functional role in the process of neural aging observed in this model, and also suggests that Snca acts as a positive regulator of stem cell maintenance.

## Introduction

Neurogenesis continues during adulthood in two brain neurogenic niches, the dentate gyrus of the hippocampus and the subventricular zone (SVZ) adjacent to lateral ventricles. In these areas new neurons are produced throughout life from stem cells, which are radial glia-like cells, expressing glial fibrillary acidic protein (GFAP), nestin and Sox2, and named in the dentate gyrus as type-1 cells (Seri et al., 2001; Filippov et al., 2003; Kronenberg et al., 2003; Komitova and Eriksson, 2004; Kempermann, 2015; Lim and Alvarez-Buylla, 2016). Stem cells of the dentate gyrus are a heterogeneous population that is mainly quiescent and does not undergo continuous rounds of division. In fact, stem cells exit quiescence and enter the cell cycle only after they are activated by a neurogenic signal, and either self-renew into quiescent stem cells or develop into proliferating progenitor cells (Artegiani et al., 2017; see for review Urbán et al., 2019; Ceccarelli et al., 2020). Progenitor cells, named type-2 and type-3, are instead prone to divide until they become early post-mitotic neuron (stage 5; Kempermann et al., 2004).

Adult neurogenesis is functionally important, as it has been demonstrated that the new neurons generated are necessary for the hippocampal dependent processes of learning and memory; in particular, the addition of new neurons to the existing circuits potentiates the ability of the dentate gyrus to separate specific memory patterns (i.e., pattern separation) (Farioli-Vecchioli et al., 2008; Aimone et al., 2011; Sahay et al., 2011; Tirone et al., 2013). While the occurrence of adult neurogenesis is evident in the mouse and rat, in humans it is still under debate, with negative (Sorrells et al., 2018) as well as positive evidence (Boldrini et al., 2018) obtained from autoptic samples.

During aging neurogenesis decreases, with reduced numbers of stem and progenitor cells as well as of new neurons generated (Kuhn et al., 1996; Bizon and Gallagher, 2003; Rao et al., 2006; Micheli et al., 2018). This decrease of neurogenesis brings a decline of the hippocampus-dependent learning and memory performances (van Praag et al., 2005; Couillard-Despres et al., 2009).

The *Btg1* gene is required to maintain the quiescence of stem and progenitor cells of the dentate gyrus and SVZ, as indicated by the observation that ablation of Btg1 causes an early postnatal increase of the proliferation of stem and progenitor cells of the dentate gyrus and SVZ, with reduced numbers of quiescent cells (Farioli-Vecchioli et al., 2012). However, this is followed by an age-dependent rapid reduction of the proliferative capability and an increased entry into quiescence of stem/progenitor cells, already detectable in young adult mice (2 months of age), further accompanied by an increase of expression of p53 and p21 in neural cells of the hippocampus, and by an increase of apoptosis (Farioli-Vecchioli et al., 2012). This strongly suggests that the *Btg1* knockout (KO) is a model for early aging of the neurogenic niches. Consistently, adult *Btg1* KO mice show impaired hippocampus-dependent associative memory (contextual fear-discrimination learning), which is however rescued by voluntary running (Farioli-Vecchioli et al., 2014).

In fact, physical exercise (i.e., running) is a powerful activator of adult neurogenesis in the dentate gyrus (van Praag et al., 1999; Vivar et al., 2016). Running triggers a rapid increase of proliferation of the neural progenitor cells, as early as after 3 days of exercise (Patten et al., 2013). It has been shown that the increase of proliferation of progenitor cells leads to the generation of new neurons that are integrated into the existing memory circuits (Vivar and van Praag, 2013), and it induces an enhancement of performance in hippocampus-dependent memory and learning tasks, including spatial memory and spatial pattern separation (Fordyce and Farrar, 1991; Creer et al., 2010), contextual fear conditioning (Kohman et al., 2012), and novel object recognition (Bolz et al., 2015).

Interestingly, voluntary running in aged mice is able not only to rescue, at least partially, the deficit of neurogenesis in terms of neuron number and morphology (i.e., synaptic connectivity), but also to counteract the spatial memory deficit (van Praag et al., 2005; Marlatt et al., 2012; Siette et al., 2013).

We have previously demonstrated that in the *Btg1* KO model of neural aging running rescues the reduced proliferation of stem/progenitor cells, by enhancing it far above the wild-type (WT) levels in stem (type-1, GFAP^+^/Sox2^+^) and progenitor cells (type-2 and type-3, DCX^+^), and rescues also the reduced associative memory (Farioli-Vecchioli et al., 2014).

On this basis, we aimed at identifying the genes and the biological processes and pathways responsible for the aging phenotype and involved in the reactivation of the prematurely aged stem cells of the *Btg1* KO model.

Thus, we subjected 2-month-old *Btg1* WT and KO mice to a running schedule of 12 days. Thereafter, we isolated their dentate gyrus and extracted the mRNA analyzing the whole transcriptome gene expression by RNA sequencing.

We found that the expression level of a number of genes in the dentate gyrus of *Btg1* KO sedentary mice was significantly changed – mainly reduced – relative to sedentary WT mice; moreover, running was able to restore in the *Btg1* KO mice the original expression of these genes. These genes, counter-regulated by running vs. the *Btg1* KO, may represent the transcriptomic signature underlying the running-induced reactivation of stem and progenitor cells in the *Btg1* KO model of reduced proliferative capability. Among these genes, *Snca* was strongly down-regulated by *Btg1* deletion. We further demonstrated that a virus-mediated expression of alpha-synuclein (Snca) in the dentate gyrus of *Btg1* KO mice is by itself able to rescue the defective proliferation of stem cells and the excess of apoptosis. This indicates that Snca plays a functional role in the process of neural aging observed in this model and in stem cell self-renewal.

## Materials and Methods

### Mouse Line, Genotyping, and Husbandry

The *Btg1* KO and WT mouse strains in the C57BL/6 background were generated as previously described (Farioli-Vecchioli et al., 2012). Genotyping was routinely performed by PCR analysis, using genomic DNA from tail tips as described (Farioli-Vecchioli et al., 2012).

Mice were maintained under standard specific-pathogen-free conditions and were housed in standard cages until P60. Then, mice were randomly assigned to running wheel or standard cages for 12 days. Wheel rotations were recorded daily with an automatic counter. After 12 days, mice were euthanized to dissect the dentate gyrus. The average running wheel distance over the whole experiment (12 days) was 7.55 km/day ± 0.54 (SEM) for WT mice and 7.17 km/day ± 1 (SEM) for KO mice, without significant differences (*p* = 0.75, Student’s *t*-test); the total distances run were on average 90.56 km ± 6.53 (SEM) for WT and 86.06 km ± 12.01 (SEM) for KO mice (*p* = 0.75, n WT mice = 10, n KO mice = 10, Student’s *t*-test). 15-month-old mice were subjected to the same protocol. All animal procedures were performed on male mice and completed in accordance with the current European (directive 2010/63/EU) Ethical Committee guidelines and the protocol of the Italian Ministry of Health (authorization 442-2016-PR). *Btg1* KO mice are available upon request to J.P. Rouault.

### Dentate Gyrus Dissection and RNA Isolation

A 2-month-old Btg1WT and KO mice were sacrificed by rapid decapitation. The isolation of bilateral dentate gyrus was performed under a stereomicroscope, either in sedentary mice or at the end of the 12-day running protocol, following a described procedure (Hagihara et al., 2009).

Dissected tissues were immediately homogenized in TRIzol Reagent (Invitrogen, San Diego, CA, United States) and total RNA extraction was performed as described previously (Farioli-Vecchioli et al., 2012). Extracted RNA was quantified and assessed for purity using a NanoDrop ND-1000 Spectrophotometer (Thermo Fisher Scientific, Wilmington, DE, United States) and an Agilent 2100 bioanalyzer (Agilent Technologies, Santa Clara, CA, United States). RNAs were subsequently employed for Transcriptome sequencing and/or for real-time PCR experiments. The identity of the dissected dentate gyri was checked by comparing the relative expression of specific markers, i.e., *Tdo2*, *Dsp*, *Meis2*, *Tyro3*, as indicated in Hagihara et al. (2009) (data not shown). The same procedure was employed to analyze the dentate gyrus of 15-month-old Btg1 WT mice, submitted to exercise or sedentary.

### Transcriptome Sequencing

For RNA-sequencing we used total RNA isolated from the dentate gyrus of *Btg1* WT or KO mice, either sedentary or submitted to running. Five samples were used in total for each of the four experimental groups, and each sample was obtained by pooling together dentate gyri of two mice.

Purified RNA was delivered to IGA Technology Services^1^ for RNA library preparation (by Illumina TruSeq Stranded mRNA Sample Prep kit, following the manufacturer’s instructions), RNA sequencing (Illumina HiSeq2500; 50 bp single-end reads, 6-plex run, 30 M reads) and standard bioinformatic analysis. Briefly, the CASAVA 1.8.2 version of the Illumina pipeline was used to process raw data for both format conversion and de-multiplexing. Reads were aligned on the mm10 genome reference assembly using TopHat/Bowtie tool, transcripts counts were performed via Cufflinks, while the pairwise differential expression analysis of gene transcripts were determined via Cuffdiff in the form of FPKM values (Trapnell et al., 2013; Ghosh and Chan, 2016). Furthermore, the RNA sequencing datasets are deposited at the Gene Expression Omnibus (GEO) repository with Accession Numbers GSE179081^2^.

### Gene Ontology Enrichment

Gene Ontology enrichment analysis was performed in order to identify GO terms significantly over-represented in genes deregulated in specific comparisons and, as a result, to suggest possible functional characteristics of these genes. Enriched GO terms in the set of genes that are significantly over-expressed or under-expressed in a specific condition may suggest possible mechanisms of regulation or functional pathways that are, respectively, activated or repressed in that condition. As a first step, we built gene and GO term associations considering the known GO annotations for both *Homo sapiens* and *Mus musculus* organisms. In detail, GO Annotations for the gene products of both organisms were downloaded from the GO Consortium web site^3^. The GO terms common to both organisms were associated to the union of the lists of the organism-specific annotated genes and the terms exclusive for one of the organisms were considered together with their specific annotated genes. The genes assayed by RNA-seq in our study were annotated by using their associations with both the GO terms common and exclusive for the two organisms. The *p*-Values for enrichment were calculated by Fisher’s exact test by using MATLAB analysis code.

### Real-Time PCR

To validate RNA sequencing results, total RNA extracted from isolated dentate gyri was reverse-transcribed as previously described (Farioli-Vecchioli et al., 2012). In each of the four groups, two of the samples analyzed were the same already employed for RNA seq and two were from new extractions. Each sample consisted of dentate gyri from two mice.

Real-time PCR was carried out with a 7900HT System (Applied Biosystems) using SYBR Green I dye chemistry in duplicate samples. Relative quantification was performed by the comparative cycle threshold method (Livak and Schmittgen, 2001). The mRNA expression values were normalized to those of the TATA-binding protein gene used as endogenous control. One *Btg1* WT sedentary mice was randomly chosen as control calibrator. Average ± SEM values of fold-changes relative to the control sample are shown. Specific RT-PCR primers used were deduced from published murine cDNA sequences and are listed in Supplementary Table 1.

### *Alpha-Synuclein* Promoter Cloning and Activity

A 2254 nucleotide sequence of DNA immediately upstream of the translation initiation codon of the mouse *Snca* gene was cloned into the 5′-*Mlu*I and 3′-*Bgl*II sites of the pGL3 basic vector, containing the sequence coding for the luciferase reporter gene, thus generating pGL3-prSnca-LUC. This promoter sequence corresponds to the active promoter region of *Snca* (Duplan et al., 2016) and was synthesized by Eurofins MWG (Ebersberg, Germany). All the constructs were verified by full sequencing. Transient co-transfections in HEK293T cells of pGL3-prSnca-LUC with a vector expressing Btg1 (pSCT-Btg1 or pSCT-empty; Ceccarelli et al., 2015) were carried out using lipofectamine (Invitrogen, San Diego, CA, United States) according to the manufacturer’s instructions. We included in all transfections the pRL-TK control reporter (Renilla luciferase driven by the thymidine kinase promoter). Luciferase assays were performed by the Dual-Luciferase reporter assay system (Promega, Madison, WI, United States) 48 h after transfection, according to the manufacturer’s instructions, as described previously (Micheli et al., 2011). We normalized the luciferase activity of each sample (L) for differences in transfection, by assaying the expression of Renilla luciferase (R) in each transfected cell extract. The normalized activity of the reporter gene was thus obtained as L/R. The fold activity was then obtained as ratio between each average value of the normalized reporter activity and the average normalized reporter activity of the corresponding control culture. Student’s *t*-test on normalized reporter activity values was used for statistical analysis.

### Generation of Recombinant *Snca*-Expressing Lentiviruses and Infection *in vivo*

The lentiviral vector pCCL-sin-PPT.hPGK.IRES.eGFP.Wpre, kindly provided by L. Naldini (see Dull et al., 1998) was used to express the cDNA sequence of mouse *Snca* in dentate gyrus cells. The construct pCCL-sin-PPT.hPGK.IRES.eGFP.Wpre-Snca was generated by cloning the optimized cDNA sequence of *Snca* in the *Bgl*II/*Xba*I sites of the lentiviral vector. The Snca sequence was synthesized by the Eurofins MWG (Ebersberg, Germany) and verified by sequencing.

The G glycoprotein vesicular stomatitis virus-pseudotyped lentiviral particles were generated by CaPh transfection of HEK293T cells with a mixture of either pCCL-sin-PPT.hPGK.IRES.eGFP.Wpre (pCCL-empty, control vector) or pCCL-sin-PPT.hPGK.IRES.eGFP.Wpre-Snca (pCCL-Snca) lentiviral vector and the three plasmids pMDL, pRSV-REV, and pVSV-G (kindly provided by L. Naldini; Dull et al., 1998) required to produce lentiviral particles. Cells were cultured in Dulbecco’s modified Eagle’s medium (DMEM) supplemented with 10% fetal bovine serum, 100 U/mL penicillin G, and 100 μg/mL streptomycin at 37°C in 5% CO_2_. 4 × 10^6^ cells were plated in a 10-cm dish 24 h before transfection. Virus-containing medium was harvested 48 and 60 h after transfection, and concentrated by two ultracentrifugation steps. The titers of the viral vectors were in the range of 108 TU/ml. The expression of transduced Snca was verified by real-time PCR and Western blot of RNA and protein from HEK293T infected cells (data not shown). The concentrated virus solution was infused (1.5 μl at 0.2 μl/min) by stereotaxic surgery in the right and left dentate gyrus of P60 *Btg1* WT and KO mice (anteroposterior = −2 mm from bregma; lateral = 1.5 mm; ventral = −2.0 mm). Mice were euthanized after 5 days.

Lentiviruses generated were replicant-deficient. Their manipulation and stereotactic injection in mice were approved by the Italian Ministry of Health (authorization RM/IC/Op2/20/005) and performed using BSL-2 and ABSL-2 containment.

### Immunohistochemistry

Brains were collected after transcardiac perfusion with 4% PFA in PBS 1x and kept overnight in 4% PFA. Brains were then equilibrated in 30% sucrose and cryopreserved at −80°C. Immunohistochemistry was performed on serial free-floating coronal sections cut at 40 μm thickness in a cryostat at −25°C from brains embedded in Tissue-Tek OCT (Sakura Finetek, Torrance, CA, United States). Sections were previously permeabilized with 0.3% TritonX-100 in PBS, and then incubated with primary antibodies with 3% normal donkey serum in 0.3% TritonX-100 in PBS for 16-18 h at 4°C.

Proliferating stem and progenitor cells infected with lentiviruses were visualized by means of a rabbit monoclonal antibody against Ki67 (Invitrogen, San Diego, CA, United States; MA514520; 1:200), and a goat polyclonal antibody against Sox2 (Abcam, Cambridge, United Kingdom; Ab239218; 1:300) and a mouse monoclonal antibody against GFAP (Sigma–Aldrich, St Louis, MO, United States; G6171; 1:200). Apoptotic cells were identified with a rabbit polyclonal antibody against cleaved (activated) Caspase-3 (Cell Signaling Technology, Danvers, MA, United States; 9661; 1:100). GFP-positive cells generated by the lentiviral construct were directly visualized by fluorescence microscopy.

Secondary antibodies used to visualize the antigen were from Jackson ImmunoResearch (West Grove, PA, United States) as follows: a donkey anti-rabbit antiserum conjugated to tetramethylrhodamine isothiocyanate (TRITC) (Ki67 and Caspase-3) and a donkey anti-goat antiserum conjugated to Alexa-647 (Sox2), both incubated for 1 h; a donkey anti-mouse antiserum conjugated to DyLight 405 (GFAP) incubated for 5 h. Nuclei were counterstained by Hoechst 33258 (Sigma–Aldrich, St. Louis, MO, United States; 1 μg/ml in PBS).

For quadruple labeling (Ki67, Sox2, GFAP, and GFP), immunofluorescence for antibodies against Ki67 and Sox2 was performed as described above, and sections were post-fixed with 4% PFA for 10 min. Then, we proceeded with incubation with the anti-GFAP antibody with 3% normal donkey serum in 0.3% TritonX-100 in PBS for 16-18 h at 4°C, followed by incubation with the secondary antibody.

In negative-control sections, the primary antibodies were omitted, to exclude non-specific signal.

Confocal Z-stacks and single plane-images of the immunostained sections were obtained using a TCS SP5 confocal laser scanning microscope (Leica Microsystem, Wetzlar, Germany).

### Quantification of Cell Numbers

Cells expressing the indicated markers were counted throughout the whole rostrocaudal extent of the dentate gyrus, by analyzing with confocal microscopy one-in-six series of 40-μm free-floating coronal sections (240 μm apart). The total estimated number of positive cells within the dentate gyrus was obtained by multiplying the average number of positive cells per section by the total number of 40-μm sections including the entire dentate gyrus (about 50-60 sections), as described (Jessberger et al., 2005; Farioli-Vecchioli et al., 2008; see also about the cell counting theory: Noori and Fornal, 2011). Therefore, about 8-10 sections (16-20 dentate gyri) per mouse and four animals per group were analyzed. Cell number analyses were performed manually by trained experimenters, in blinded fashion, using the IAS software to register positive cells (Delta Sistemi, Rome, Italy).

### Statistical Analyses

Analysis of pairwise comparison of differential gene expression, i.e., the comparison of the mean fold-expression changes between samples of two groups from the different data sets (*n* = 5 samples per group), was performed with Cuffdiff *p*-Value (*p*), corrected for False Delivery Rate to obtain the *q*-Value (*q*) (Figures 3, 4).

**FIGURE 1:**
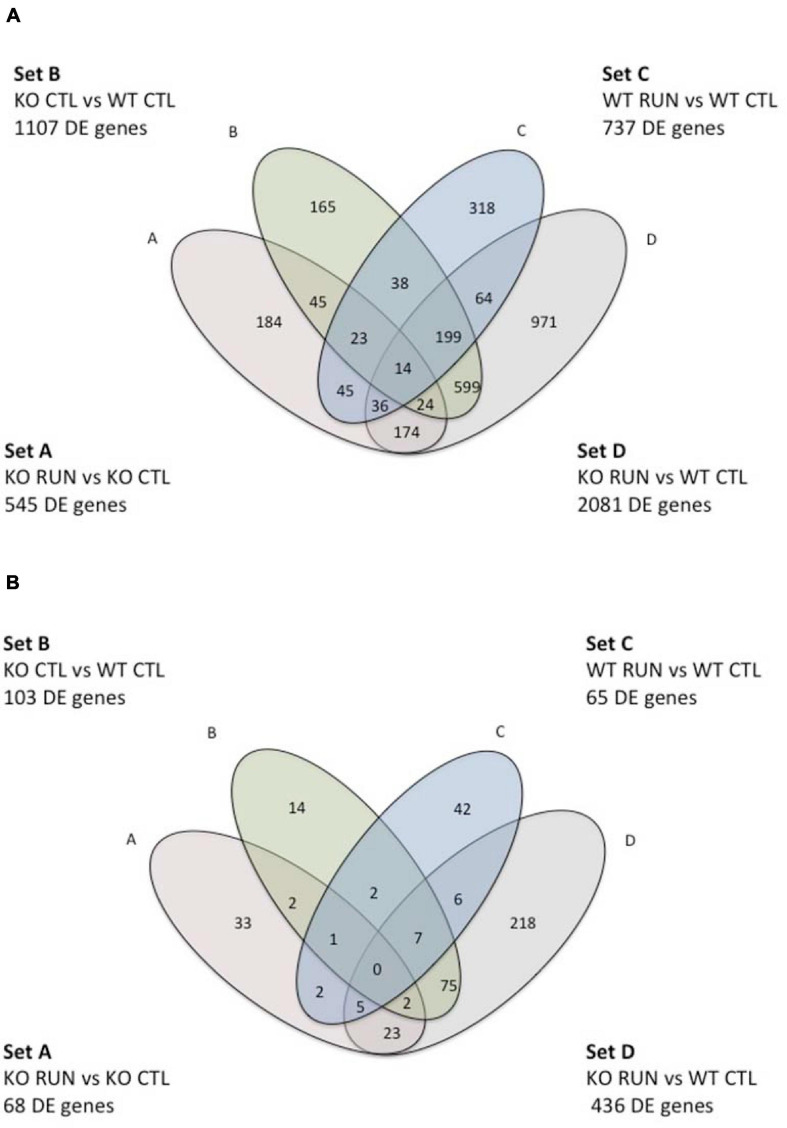
Venn Diagram indicating pairwise comparisons of four genotypes and the intersection of their differentially expressed gene sequences in sets A-D. In the upper and lower figure, Set A corresponds to the pairwise comparison Btg1 KO RUN vs. Btg1 KO CTL; set B represents Btg1 KO CTL vs. Btg1 WT CTL; set C indicates Btg1 WT RUN vs. Btg1 WT CTL, and set D corresponds to the comparison Btg1KO RUN vs. Btg1 WT CTL. The upper diagram **(A)** is referred to genes differentially expressed in each set with *p*-Value < 0.05; the lower diagram **(B)** is referred to genes differentially expressed in each set with *q*-Value < 0.05 (*q*-Value is the *p*-Value adjusted to control the False Discovery Rate).

**FIGURE 2:**
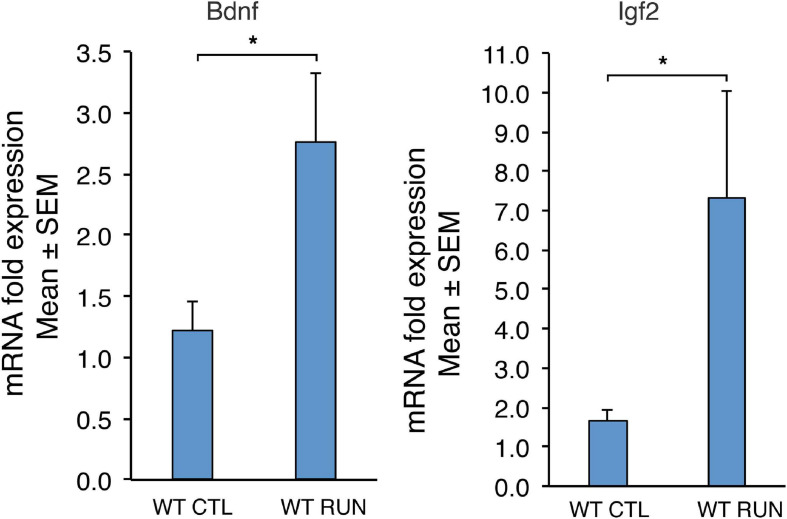
Validation by real-time PCR in the dentate gyrus, isolated from 2-month-old mice, of two genes up-regulated by running (*Bdnf* and *Igf2*) among the 52 genes common between set C (sedentary Btg1 wild-type vs. running *Btg1* wild-type mice) and the data sets of Grégoire et al. (2018). For WT CTL and WT RUN, the average mRNA fold expression ± standard error of the mean (mean ± SEM) is plotted. Mean ± SEM fold increases are from three independent experiments. TBP was used to normalize data. *p < 0.05, Student’s *t*-test.

**FIGURE 3:**
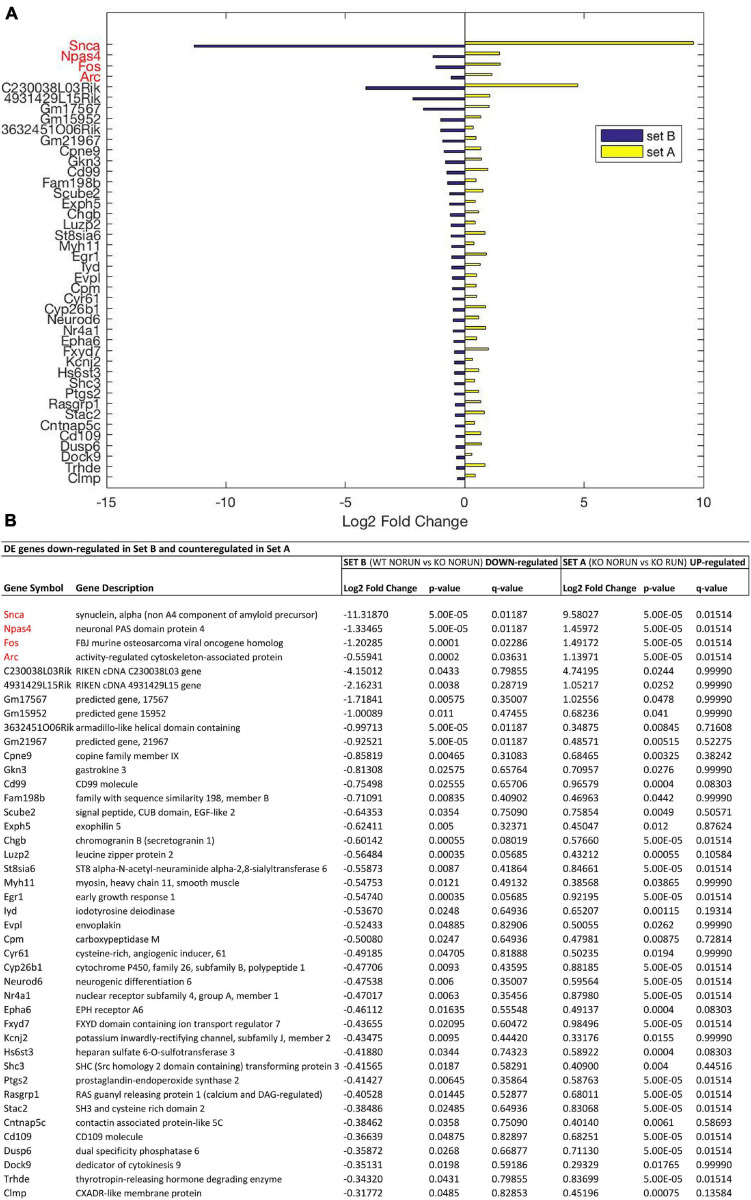
Genes down-regulated by *Btg1* knockout and counter-regulated by running. **(A)** Bar plot showing the log2 fold change values determined by RNA-seq for the genes which are significantly reduced in Btg1 KO CTL relative to WT CTL (set B; in blue) and increased in Btg1 KO RUN relative to Btg1 KO CTL (set A; in yellow). **(B)** The table presents for each gene its description together with the log2 fold change, the *t*-test *p*-Value and *q*-Value in both set A and set B comparisons. The pairwise comparisons between the means of the two groups of each set were performed by the Cuffdiff software. In order to overcome the multiple testing problem, the resulting *p*-Values were adjusted to obtain *q*-Values for False Discovery Rate (IGA software; *n* = 5 per group). All the genes presented in the figure panels are differentially expressed in both sets with *p*-Value < 0.05, whereas the genes in red have also a *q*-Value < 0.05 in both sets A and B. The genes are sorted in descending order of the absolute value of their log2 fold changes, starting with the four genes presenting *q* < 0.05 in both sets.

**FIGURE 4:**
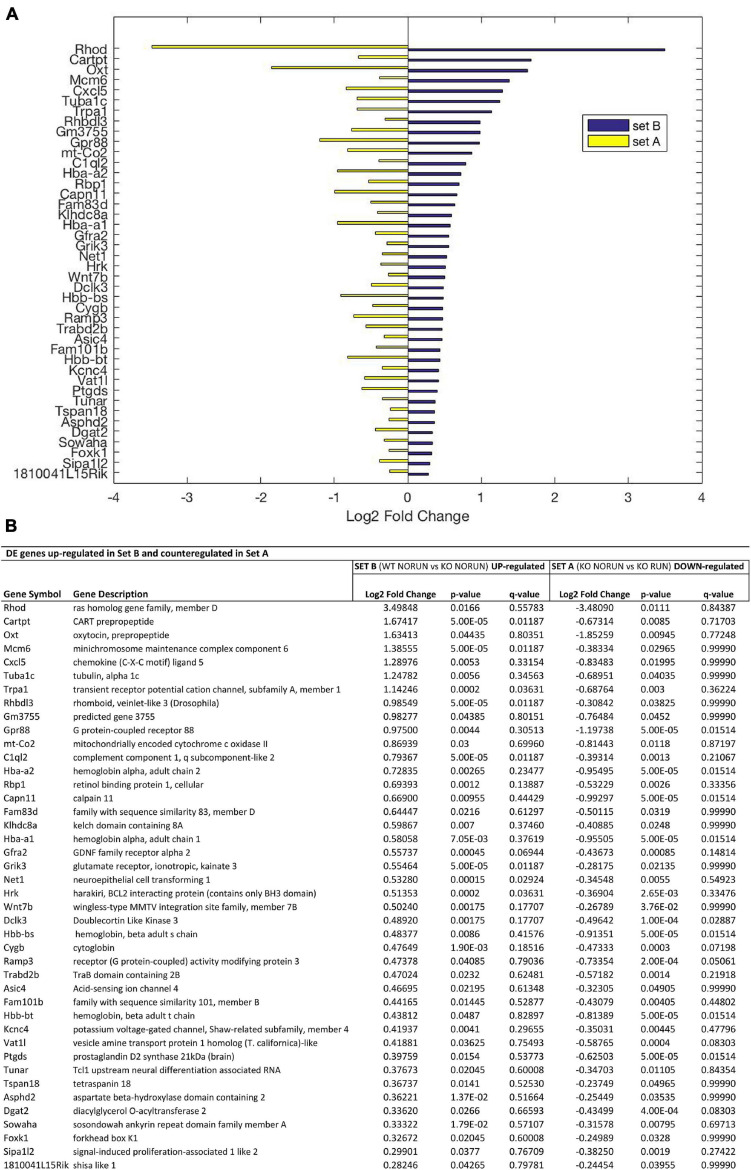
Genes up-regulated by *Btg1* knockout and counter-regulated by running. **(A)** Bar plot showing the log2 fold change values determined by RNA-seq for the genes which are significantly increased in Btg1 KO CTL relative to WT CTL (set B; in blue) and reduced in Btg1 KO RUN relative to Btg1 KO CTL (set A, in yellow). **(B)** The table presents for each gene its description together with the log2 fold change, the *p*-Value and *q*-Value in both set A and set B comparisons (IGA software; *n* = 5 per group). All the genes presented in panels **(A,B)** are differentially expressed in both sets with *p*-Value < 0.05, and are sorted in descending order of their log2 fold changes.

The statistical significance of the overlap between differentially expressed (DE) genes belonging to different data sets (Table 1), or the enrichment probability of the DE genes in each GO functional class (Table 2 and Supplementary Figures 1, 3, 4), were calculated using the Fisher’s Exact test.

**TABLE 1:** Genes activated by running in dentate gyrus, common between DE genes of set C with *p*-Value ≤ 0.05 and DE genes of Grégoire et al. (2018) study.

		**Set C**	**RUN**	**HRUN**	**LRUN**
**Fisher’s exact test, *p* vs. Set C**			**4.7 × 10^–7^**	**9.4 × 10^–19^**	**2.5 × 10-4**
**Ensemble ID**	**Gene symbol**	**Log2 fold change**	**Log2 fold change**	**Log2 fold change**	**Log2 fold change**
ENSMUSG00000029361	NOS1	−0.470375	−0.578469652	−0.453798216	−0.737960989
ENSMUSG00000004151	ETV1	−0.300321	−0.304629088	−0.317303481	*
ENSMUSG00000021478	DRD1	0.865092	0.354327344	0.432724935	*
ENSMUSG00000029819	NPY	0.32182	*	*	0.31682607
ENSMUSG00000060534	DCC	−0.382755	*	*	−0.335588201
ENSMUSG00000039579	GRIN3A	−0.294909	−0.301258838	−0.33259443	−0.31771898
ENSMUSG00000048482	BDNF	0.294908	*	0.361710666	*
ENSMUSG00000032128	ROBO3	0.412667	0.459296473	0.426910508	0.524813209
ENSMUSG00000048583	IGF2	0.56748	0.574986147	*	1.095855535
ENSMUSG00000054640	SLC8A1	−0.560071	*	*	−0.40849222
ENSMUSG00000039323	IGFBP2	0.833008	*	*	0.387501516
ENSMUSG00000041695	KCNJ2	0.384918	*	0.387747369	*
ENSMUSG00000019929	DCN	−1.12184	*	−0.5223705	*
ENSMUSG00000033685	UCP2	0.786285	*	*	0.303431949
ENSMUSG00000026834	ACVR1C	0.551476	*	0.31216698	*
ENSMUSG00000023034	NR4A1	−0.65185	*	*	−0.34630094
ENSMUSG00000046743	FAT4	−0.281594	−0.414200001	−0.419286349	−0.334485522
ENSMUSG00000090125	POU3F1	−0.811238	*	*	−0.420374444
ENSMUSG00000026278	BOK	0.940528	*	0.410005165	*
ENSMUSG00000043631	ECM2	−0.725189	−0.308866264	−0.333604481	*
ENSMUSG00000039206	DAGLB	0.402413	*	0.335848283	*
ENSMUSG00000053310	NRGN	0.451917	*	0.322949175	0.300507274
ENSMUSG00000022602	ARC	−0.541134	−0.334167922	*	−0.453081688
ENSMUSG00000059991	NPTX2	0.432027	0.324387216	0.419791099	*
ENSMUSG00000037984	NEUROD6	−0.360006	*	*	−0.339904424
ENSMUSG00000056596	TRNP1	0.327255	0.31915195	0.400566672	*
ENSMUSG00000079056	KCNIP3	0.336908	0.316770424	*	*
ENSMUSG00000036578	FXYD7	0.365723	*	0.345230774	*
ENSMUSG00000029843	SLC13A4	0.955388	0.432078952	*	0.827053713
ENSMUSG00000031343	GABRA3	−0.438132	*	−0.402627901	*
ENSMUSG00000073565	PRR16	−0.83169	*	−0.301348939	*
ENSMUSG00000050074	SPINK8	−1.84603	*	*	−0.30319055
ENSMUSG00000027674	PEX5L	−0.374298	*	*	−0.403219466
ENSMUSG00000023046	IGFBP6	0.903935	0.454044535	0.458461297	0.557625529
ENSMUSG00000036357	GPR101	−0.99491	*	−0.304377347	*
ENSMUSG00000029816	GPNMB	0.929795	0.323659965	0.355055933	*
ENSMUSG00000031557	PLEKHA2	0.836955	0.410647957	0.456199366	0.336819784
ENSMUSG00000039457	PPL	0.423926	0.429833239	0.385231614	0.442158916
ENSMUSG00000021182	CCDC88C	−0.584561	*	*	−0.387131468
ENSMUSG00000024558	MAPK4	0.406694	0.306824378	*	*
ENSMUSG00000075334	RPRM	0.72366	0.53557688	0.511042639	0.53753504
ENSMUSG00000035202	LARS2	1.40407	*	0.420399217	0.483818263
ENSMUSG00000042109	CSDC2	0.47945	0.327925685	0.30461975	0.340936421
ENSMUSG00000090546	CDR1	−0.516516	−0.301073404	*	−0.384470606
ENSMUSG00000040794	C1QTNF4	0.346179	*	*	0.358255201
ENSMUSG00000075702	SELM	0.495742	*	*	0.322852208
ENSMUSG00000066607	INSYN1	0.506255	*	0.315879504	*
ENSMUSG00000090291	LRRC10B	0.966886	*	0.405425364	*
ENSMUSG00000013367	IGLON5	0.591971	0.335604792	0.392746828	0.409496208
ENSMUSG00000041708	MPPED1	−0.50187	*	*	−0.331815189
ENSMUSG00000079065	BC005561	−0.40994	*	*	−0.441620487
ENSMUSG00000026185	IGFBP5	−0.337166	−0.501634451	−0.550599528	−0.482583848

Genes whose expression is up- or down-regulated by running in wild-type Btg1 dentate gyrus (Set C, sedentary *Btg1* wild-type vs. running *Btg1* wild-type group), and that correspond to genes whose expression was described to be changed by running in the study by Grégoire et al. (2018). The overlap between DE genes belonging to the Set C and to the three subgroups shown in the study of Grégoire et al. (2018), i.e., RUN, or with high (H-RUN) and low running performance (L-RUN), resulted highly significant (*p*-Values are calculated by Fisher’s exact test, see the top row in the table). All expression changes in the DE genes of set C are concordant with those of the sets of Grégoire et al. (2018). The symbol * indicates genes with *p*-Value > 0.05.

**TABLE 2:** The most representative biological processes (GO terms) enriched in DE genes counter-regulated by running in set A vs. set B (*Btg1* knockout).

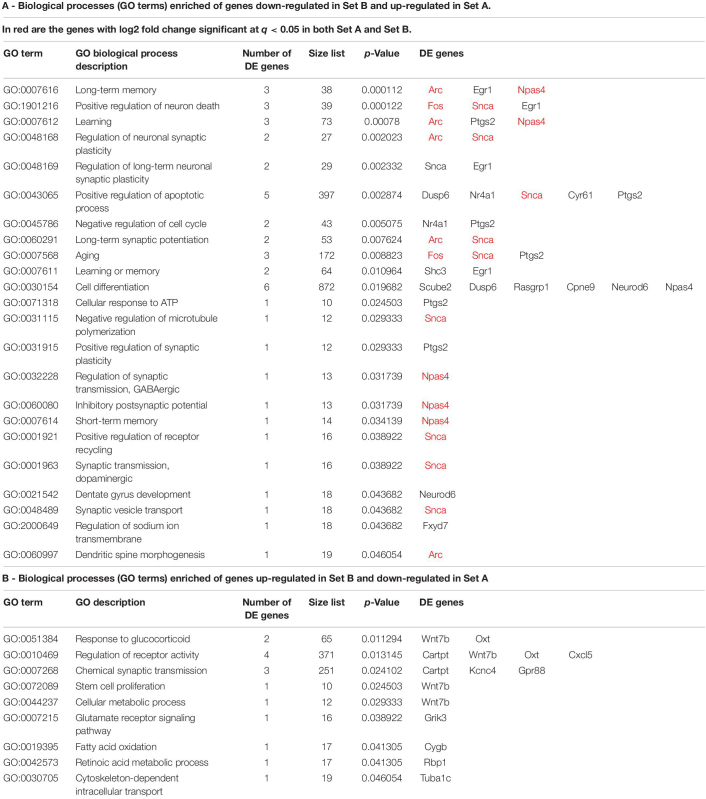

***(A)** List of biological processes enriched in genes down-regulated in Set B and up-regulated in Set A; **(B)** List of biological processes enriched in genes up-regulated in Set B and down-regulated in Set A. In red are the genes differentially expressed with significant log2 fold change at *q*-Value < 0.05 in both Set A and Set B and that, notably, are all down-regulated in set B and up-regulated in Set A; in black are differentially expressed genes with significant log2 fold change at *p*-Value < 0.05. The enrichment *p*-Value of the DE genes in each functional class has been calculated using the Fisher’s Exact test. GO term and description columns contain the identifier and the name of the GO group. Number of DE genes is referred to the genes of Set A correspondingly counter-regulated in Set B, mapping to that GO group. Size list: total number of genes belonging to the specific GO process. See in Supplementary Figures 3, 4 the full list of GO processes.*

**FIGURE 5:**
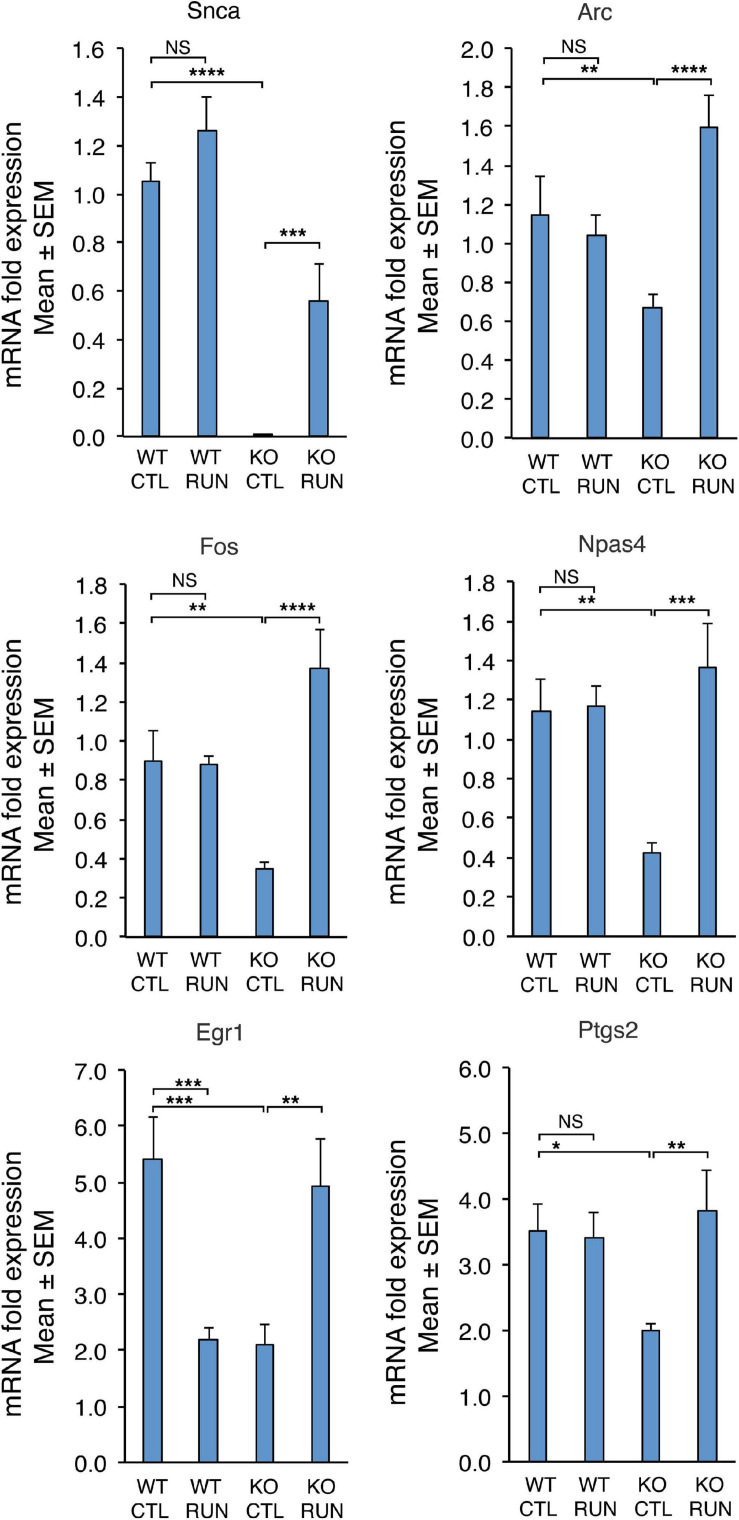
The differential expression of six genes down-regulated in Set B and counter-regulated in Set A, either with statistical significance *q*-Value < 0.05 (*Snca*, *Arc*, *Fos*, *Npas4*) or with statistical significance *p*-Value < 0.05 (*Egr1*, *Ptgs2*), was confirmed by real-time PCR in the dentate gyrus isolated from 2-month-old mice belonging to the four groups analyzed. Shown are the mean mRNA expression fold increases ± SEM from three independent experiments. Two-way ANOVA, treatment effect: *Snca F*(1,30) *p* = 0.0009, *Arc F*(1,38) *p* = 0.0041, Fos *F*(1,20) *p* = 0.0004, Npas4 *F*(1,28) *p* = 0.0039, Egr1 *F*(1,24) *p* = 0.73, and *Ptgs2 F*(1,26) *p* = 0.048. TBP was used to normalize data. *p < 0.05, **p < 0.01, ***p ≤ 0.001, ****p < 0.0001, or NS *p* > 0.05, two-way ANOVA, Fisher PLSD *post hoc* test.

**FIGURE 6:**
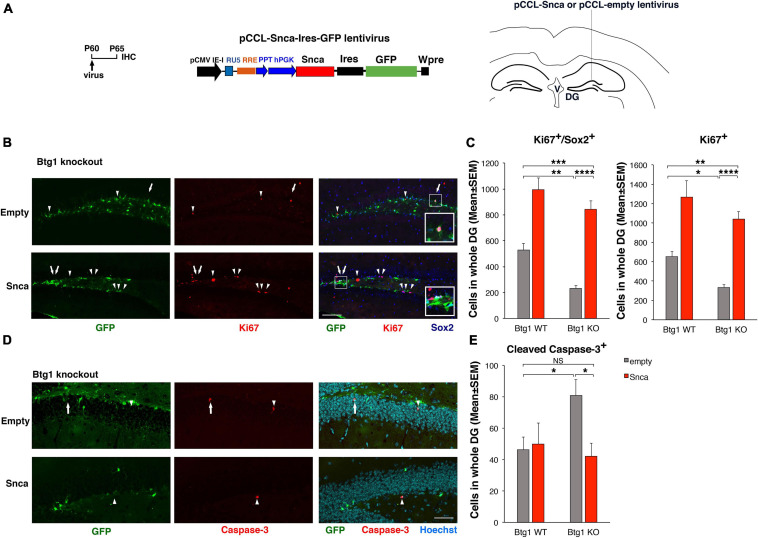
Rescue of the defective alpha-synuclein expression in *Btg1* knockout dentate gyrus through virus-mediated transfer reactivates stem cell proliferation and reverses the increased apoptosis. **(A)** Lentivirus infection protocol, structure and injection area of the virus. DG, dentate gyrus; V, ventricle. **(B)** Representative confocal images (20 × magnification) of coronal sections of the dentate gyrus of *Btg1* knockout mice, labeled with Ki67, Sox2 and GFP (in red, blue and green, respectively), 5 days after infection with either pCCL-Snca or pCCL-empty lentiviruses. The white arrows indicate triple-labeled cells for GFP, Ki67, and Sox2; white arrowheads indicate cells labeled for Ki67. The white box area is shown with 2.4 × digital magnification. Scale bars, 100 μm. **(C)** Quantification of the absolute number of proliferating dentate gyrus stem cells (type-1-2a; Ki67^+^/Sox2^+^) and of total proliferating progenitor cells (Ki67^+^). The significant decrease of Btg1 KO stem cells (infected with empty virus), relative to Btg1 WT, is reversed above control levels in KO mice infected with pCCL-Snca virus (Analysis of simple effects: *p < 0.05; **p < 0.01; ***p < 0.001; or ****p < 0.0001, Fisher’s PLSD ANOVA *post hoc* test). **(D)** Representative images by confocal microscopy (40 × magnification) of apoptotic cells in the dentate gyrus of *Btg1* knockout mice, labeled with activated Caspase-3 (in red) and GFP (in green), 5 days after infection with either GFP-Snca or GFP-empty lentiviruses. Nuclei are counterstained with Hoechst 33258 (cyan). The white arrow indicates a cell double positive for GFP and activated Caspase-3 and the white arrow heads indicate cells positive for Caspase-3. Scale bars, 50 μm. **(E)** Quantification of the absolute number of apoptotic stem cells (Caspase-3^+^). The increase of apoptosis observed in Btg1 KO dentate gyrus, relative to Btg1 WT, infected with empty lentivirus is reversed by infection with lentivirus overexpressing GFP-Snca. *p < 0.05; NS *p* > 0.05; Mann-Whitney, *U*-test. Cell numbers in the dentate gyrus in panels **(C,E)** are means ± SEM of the analysis of four animals per group.

The statistical significance of the expression data obtained by real-time PCR was evaluated by two-way ANOVA when the main effects of both genotype and running were tested (Figure 5), or by one-way ANOVA when running and age effects were analyzed together (Supplementary Figure 7); individual between-group comparisons to test simple effects were carried out by Fisher’s PLSD ANOVA *post hoc* test. Instead, Student’s *t*-test was used for analysis of real-time PCR expression data when only two groups were tested (Set C: sedentary *Btg1* WT vs. running Btg1 WT) (Figure 2). The immunohistochemical proliferation data of *Btg1* WT and *Btg1* KO dentate gyri injected with Snca or empty lentivirus were analyzed with two-way ANOVA followed by Fisher’s PLSD ANOVA *post hoc* test (Figure 6 and Supplementary Figure 6); apoptosis data presented unequal variance, as indicated by Levene’s and Bartlett’s tests, therefore simple effects were analyzed with the non-parametric Mann-Whitney *U*-test (Figure 6). These analyses were carried out using the Stat View 5.1 software (SAS Institute, Cary, NC, United States) and XLSTAT (Addinsoft, Paris, France). Differences were considered statistically significant at *p* < 0.05. Real-time PCR and immunohistochemistry data were expressed as mean ± SEM.

## Results and Discussion

### Changes in Global Gene Expression Induced by Running in the Dentate Gyrus of the *Btg1* KO Aging-Like Model

Through RNA sequencing, we examined the transcriptomic profiles of the dentate gyrus isolated from 2-month-old mice, either *Btg1* WT or *Btg1* KO with aging-like neural phenotype such as reduced proliferative capability of stem and progenitor cells, submitted to physical exercise or sedentary. The WT and KO mice were submitted to voluntary running (called here WT RUN and KO RUN) for 12 days, according to a previously used protocol (Farioli-Vecchioli et al., 2014). We checked that no significant differences in running ability occurred between the two genotypes (see Materials and Methods section Mouse cell lines, genotyping and husbandry). At the end of the 12-day exercise schedule the WT RUN and KO RUN dentate gyrus was isolated following a described procedure (Hagihara et al., 2009) and processed for transcriptomic analysis, together with the dentate gyrus from the control sedentary *Btg1* WT (WT CTL) and *Btg1* KO mice (KO CTL).

The four pairwise comparisons of DE genes presented here were performed with significance threshold of the log2 fold change of gene expression set to *p* < 0.05. The two pairwise comparisons that we took chiefly in consideration are defined as Set B and Set A, which comprise the genes whose expression is significantly changed by *Btg1* knockout, relative to *Btg1* WT (Set B: Btg1 KO CTL vs. Btg1 WT CTL, 1107 genes; Figure 1A, Venn diagram), or changed by running in *Btg1* KO mice, relative to *Btg1* KO sedentary mice (Set A: Btg1 KO RUN vs. Btg1 KO CTL, 545 genes; Figure 1A).

The other two pairwise comparisons taken into consideration are Sets C and D that include the genes with a significant change of expression triggered by running, either in *Btg1* WT compared with sedentary (Set C: Btg1 WT RUN vs. Btg1 WT CTL), or in *Btg1* KO, compared with *Btg1* WT sedentary (Set D: Btg1 KO RUN vs. Btg1 WT CTL). We found 737 DE genes belonging to Set C and 2081 genes to Set D (see Venn diagram, Figure 1A).

If we limit the significance threshold of the log2 fold expression change of DE genes to *q* < 0.05 (i.e., the *p*-Value adjusted in order to reduce the False Discovery Rate for false positive), it turns out that 103 genes belong to Set B, 68 genes to Set A, 65 DE genes to Set C and 436 genes to Set D (Venn diagram, Figure 1B).

#### Concordant Changes With Another Study of Gene Expression Modulated by Running in the Dentate Gyrus

First, we sought to verify the consistence of our system with existing data, and with this aim we compared the genes differentially expressed by running in Set C (i.e., Btg1 WT RUN vs. Btg1 WT CTL), to sets of transcripts previously described to be changed by running in the isolated dentate gyrus (Vivar and van Praag, 2013; Grégoire et al., 2018). With significance threshold set to *p* < 0.05, we found that of the 737 genes differentially expressed in Set C (Btg1 WT RUN vs. Btg1 WT CTL), 52 resulted differentially and concordantly regulated by running in the Grégoire et al. (2018) study. This consisted of three subgroups, i.e., RUN and L-RUN and H-RUN, the latter two including DE gene profiles of the dentate gyrus from mice with the lowest and highest running performance, respectively (Table 1).

The overlap between DE genes belonging to the Set C and to the three subgroups of the study of Grégoire et al. (2018) resulted significantly greater than by chance, with a *p*-Value calculated by Fisher’s exact test (*p* = 4.7 × 10^–7^, 9.4 × 10^–19^, and 2.5 × 10^–4^ for the RUN, H-RUN and L-RUN groups, respectively, vs. set C; Table 1).

The 52 genes induced by running, common to set C and to the sets by Grégoire et al. (2018), resulted enriched in GO biological processes, e.g., neurogenesis and synaptic plasticity (*Nos1*, *Bdnf*, *Grin3a*, *Nrgn*, and *Arc*), regulation of cell proliferation (*Igf2*, *Igfbp2*, and *Igfbp5*), neuron development and differentiation (*Neurod6* and *Robo3*), and neurotransmitter signaling (*Npy*) (see Supplementary Figure 1). All these processes are known to be induced by running (for review: Cooper et al., 2018; Micheli et al., 2018).

By real-time PCR we also confirmed the changes of expression of *Bdnf* and *Igf2* -well known running-inducible genes- in set C, which resulted differentially regulated with statistical significance *p* < 0.05 (Figure 2; set C: Btg1 WT CTL vs. Btg1 WT RUN: *Bdnf p* = 0.042 *n* = 8, *Igf2 p* = 0.017 *n* = 16; Student’s *t*-test).

Thus, the overall significant concordance in DE transcripts identified as induced by running in our RNA-seq data and in the analysis by Grégoire et al. (2018), supports the validity of our experimental data.

### Identification of Genes Differentially Expressed in *Btg1* KO (in Set B) and Counter-Regulated by Running (in Set A)

This report focuses on the DE genes that, in sedentary mice, are significantly altered by *Btg1* deletion, relative to *Btg1* WT (Set B: Btg1 KO CTL vs. Btg1 WT CTL; Figure 1) and on the DE genes that are changed by running in *Btg1* KO mice, relative to *Btg1* KO sedentary mice (Set A: Btg1 KO RUN vs. Btg1 KO CTL; Figure 1).

In particular our interest is addressed to identify genes whose expression is modified by *Btg1* knockout (Set B) and whose change is reversed by running (Set A). These DE genes should be associated to the phenotype of early neural aging and its recovery as well as to the control of stem cell quiescence. Setting the threshold of significance at *p* < 0.05, we identified, as mentioned above, 1107 genes in Set B whose expression was significantly changed by *Btg1* knockout (either up- or down-regulated), and 545 genes in Set A changed by running. Of all these genes, 42 were down-regulated in Set B, i.e., by *Btg1* knockout, and correspondingly up-regulated in Set A, by running (Figure 3 and Supplementary Figure 2). These genes comprise among them *Arc*, *Egr1*, *Epha6*, *Fos*, *Fxyd7*, *NeuroD6*, *Npas4*, *Snca*, which are involved in synaptic plasticity, memory and aging (Chauhan and Siegel, 1996 [*Fxyd7*]; Liu et al., 2004 [*Snca*]; Uittenbogaard et al., 2010 [NeuroD6]; Mo et al., 2015 [*Egr1*]; Minatohara et al., 2016 [*Fos*]; Sun and Lin, 2016 [*Npas4*]; Nikolaienko et al., 2018 [*Arc*]; Petschner et al., 2018 [*Epha6*]). Furthermore, another 42 genes resulted up-regulated in Set B, i.e., by *Btg1* knockout, and correspondingly down-regulated in Set A, by running (Figure 4 and Supplementary Figure 2). These genes include *Wnt7b*, *Grik3*, *Gpr88*, involved in stem cells proliferation, dendritic growth, signaling and learning (Papachristou et al., 2014; Benes, 2015 [*Grik3*]; Meirsman et al., 2016 [*Gpr88*]; Ferrari et al., 2018 [*Wnt7b*]).

Moreover, if we select the genes differentially regulated with higher statistical significance (*q* < 0.05), among all the *Btg1* KO-modified genes (set B) also significantly counter-regulated by running (Set A), only four genes emerged that were all down-regulated by *Btg1* knockout and up-regulated by running (Figure 3). These DE genes were *Arc* (set B: *q* = 0.036, set A: *q* = 0.015), *Fos* (set B: *q* = 0.022, set A: *q* = 0.015), *Npas4* (set B: *q* = 0.011, set A: *q* = 0.015), and *Snca* (set B: *q* = 0.011, set A: *q* = 0.015); (Figure 3, genes written in red).

The expression changes of the four genes *Arc*, *Fos*, *Npas4*, and *Snca*, differentially regulated with statistical significance *q* < 0.05, were validated by real-time PCR in the dentate gyrus isolated from 2-month-old mice belonging to the four groups analyzed. The real-time PCR expression values of all these genes resulted significantly decreased in Set B and increased in Set A (Figure 5; set B: Btg1 KO CTL vs. Btg1 WT CTL: *Arc p* = 0.0025, *Fos p* = 0.0036, *Npas4 p* = 0.0023, *Snca p* < 0.0001; set A: Btg1 KO RUN vs. Btg1 KO CTL: *Arc p* < 0.0001, *Fos p* < 0.0001, *Npas4 p* = 0.0002, *Snca p* = 0.001; two-way ANOVA, PLSD *post hoc* test). By real-time PCR we further confirmed the expression changes of *Egr1* and *Ptgs2*, genes differentially regulated in both set B and set A with statistical significance *p* < 0.05, and known to be involved in synaptic plasticity and memory (Figure 5; set B: Btg1 KO CTL vs. Btg1 WT CTL: *Egr1 p* = 0.0004, *Ptgs2 p* = 0.0165; set A: Btg1 KO RUN vs. Btg1 KO CTL: *Egr1 p* = 0.0019, *Ptgs2 p* = 0.0046; two-way ANOVA, Fisher PLSD *post hoc* test).

### Functional Analysis of the Differentially Expressed Genes and Pathways Regulated by Running in the Dentate Gyrus of *Btg1* KO Mice

By analyzing the GO databases, we then sought to identify the biological processes that are significantly enriched in the genes either down-regulated (42) or up-regulated (42) in set B in the Btg1 KO aging model, and correspondingly counter-regulated in set A by running (Supplementary Figures 3, 4). In particular the 42 genes down-regulated in Set B and up-regulated in Set A are significantly enriched in biological processes that appear representative of the Btg1 KO phenotype and of the counter-effect of running: a selection is shown in Table 2. These processes are involved mainly in the regulation of synaptic plasticity (*Arc*, *Snca*), GABAergic synaptic transmission (*Npas4*), synaptic vesicle transport (*Snca*), dendritic spine morphogenesis (*Arc*), memory (long and short-term memory, *Npas4*, *Arc*, *Egr1*), learning (*Arc*, *Ptgs2*, *Npas4*) and long-term synaptic potentiation (*Arc*, *Snca*), aging (*Fos*, *Snca*, *Ptgs2*), cell cycle regulation (*Ptgs2/Cox2*, *Nr4A1/Nur77*) and stem cell proliferation (*Wnt7b*); see Table 2. Therefore, these GO processes impact on the dentate gyrus function of neuronal synaptic activity and plasticity, learning and memory, and on aging.

We will analyze here the biological processes enriched in genes with opposite regulation in the two Sets A and B and relevant to the *Btg1* KO phenotype, in order to interpret the transcriptomic signature of the effect of exercise on the *Btg1* KO neural aging model.

We will mainly focus on the most statistically significant DE genes, i.e., with *q* < 0.05, which presented great differential change between the two sets, and which are, notably, all down-regulated in Set B and counter-regulated in Set A (i.e., *Snca*, *Fos*, *Arc, Npas4*; Figure 3), while no DE gene with high statistical significance is up-regulated in Set B and down-regulated in Set A (Figure 4 and Table 2). This suggests that the deletion of *Btg1* is most effective in depressing the expression of specific genes while running rescues their down-regulation. We also tested whether, by restoring Snca expression in the dentate gyrus, is possible to rescue the defective processes.

#### Proliferation and Cell Cycle – Rescue by *Snca* of the Defective Proliferation in *Btg1* KO Dentate Gyrus

We have previously observed that the dentate gyrus of *Btg1* KO adult mice shows a decrease of proliferation of the stem and progenitor cells, as a consequence of the hyper-proliferation of this cellular population during the post-natal stage (Farioli-Vecchioli et al., 2012).

*Btg1* is a negative regulator of cell cycle and inhibits cyclin D1, which controls the G1 to S transition (Ceccarelli et al., 2015). In fact, here we observe that cyclin D1 expression is increased in *Btg1*-null compared to *Btg1* WT dentate gyrus (set B, log2 fold change 0.51, *p* = 0.0048). Nevertheless, *Btg1*-null stem cells in 2-month-old mice have reduced proliferative capability, a fact that may be explained by the observation that *Btg1*-null stem cells present a several-fold increase of the cyclin-dependent kinase inhibitor p21Cip1 protein, which evidently inhibits proliferation (Farioli-Vecchioli et al., 2012). Notably, running is able to completely reverse the deficit of proliferation of *Btg1* KO by inducing stem and progenitor cells to reenter the cell cycle (Farioli-Vecchioli et al., 2014). That great increase of proliferating stem cells may be associated also to the increase, observed here in set A, of the expression of the genes *Ptgs2*/*Cox2* and *Nr4a1*/*Nur77* (Figure 3, Table 2, and Supplementary Figure 3). Indeed, Ptgs2/Cox2 induces the synthesis of Prostaglandin E2 which stimulates the proliferation of neural cells through Wnt (Wong et al., 2016), and also actively stimulates the proliferation of hippocampal progenitors (Sasaki et al., 2003), whereas Nr4a1/Nur77 enhances the survival of neural cells (Xiao et al., 2013).

Moreover, *Snca* is the gene presenting the greatest decrease of expression in the Btg1 KO dentate gyrus, which is powerfully reversed by running (Figure 3). Snca is highly expressed in brain and hippocampus^4^, localized at neuron synapses, where controls clustering and release of synaptic vesicles (Bobela et al., 2015). Deregulation of Snca has been involved in the onset of neurodegenerative diseases such as Parkinson’s disease and Alzheimer’s disease (Mikolaenko et al., 2005; Bobela et al., 2015). However, the role played by Snca in many cellular processes still needs to be clarified (e.g., proliferation, apoptosis). We reasoned that a study of the significance of Snca down-regulation observed in the Btg1 KO model may provide new insights into Snca activity.

Although no GO process related to the cell cycle includes Snca, it has been observed that Snca overexpression in the neural PC12 or in SH-SY5Y neuroblastoma cells induces an increase of proliferation (Lee et al., 2003; Rodríguez-Losada et al., 2020). Rodríguez-Losada et al. (2020) show that this effect is function of its expression levels, and as a whole this suggests that Snca plays a role in cell cycle progression. Therefore, we sought to test the possibility that the rescue of the proliferative defect of *Btg1*-null dentate gyrus cells exerted by running may be achieved by reverting to normal levels the severely reduced expression of Snca. Proliferating type-1-2a stem and progenitor cells were identified in the dentate gyrus of 2-month-old *Btg1* WT and KO mice as cells marked by Ki67, which labels cycling cells (Scholzen and Gerdes, 2000), and by Sox2, which labels essentially stem cells (i.e., type-1 cells) and a fraction of type-2a progenitor cells (Filippov et al., 2003; Kronenberg et al., 2003; Komitova and Eriksson, 2004; Steiner et al., 2006). *In vivo* we infected the dentate gyrus cells of 2-month-old (P60) *Btg1*-null and *Btg1* WT mice with either a Snca-expressing or an empty lentivirus (Figure 6A), able to transduce proliferating progenitor cells as well as post-mitotic neurons (Lewis et al., 1992). Five days after injection, we observed that the proliferation of *Btg1*-null stem cells (Ki67^+^Sox2^+^) increased about 3.6-fold, relative to dentate gyrus cells injected with empty virus (two-way ANOVA, treatment effect *F*(1,243) *p* < 0.0001. Btg1 KO empty virus vs. *Btg1* WT empty virus, 57% decrease, *p* = 0.0018; Btg1 KO Snca virus vs. Btg1 KO empty virus, 3.6-fold increase, *p* < 0.0001; PLSD *post hoc* test, Figures 6B,C and Supplementary Figure 5A). Moreover, the number of proliferating Ki67^+^ Sox2^+^ cells in *Btg1* KO also exceeds the control levels (two-way ANOVA, treatment effect *F*(1,243) *p* < 0.0001. Btg1 KO Snca virus vs. Btg1 WT empty virus, 59% increase, *p* = 0.0009; PLSD *post hoc* test, Figures 6B,C and Supplementary Figure 5A). A similar activation by the Snca virus was observed for total cycling progenitor cells (Ki67^+^; two-way ANOVA, treatment effect *F*(1,243) *p* < 0.0001. Btg1 KO empty virus vs. Btg1 WT empty virus, 49% decrease, *p* = 0.030; Btg1 KO Snca virus vs. Btg1 KO empty virus, 3.1-fold increase, *p* < 0.0001; PLSD *post hoc* test, Figures 6B,C and Supplementary Figure 5A). Furthermore, we also analyzed specifically the radial glia-like proliferating stem cells (type-1; Ki67^+^GFAP^+^Sox2^+^) in dentate gyri infected with Snca virus, and we confirmed that their number is increased, relative to cells in dentate gyri injected with empty virus (two-way ANOVA, treatment effect *F*(1,140) *p* = 0.0077; Btg1 KO Snca virus vs. Btg1 KO empty virus, 2.5-fold increase, *p* < 0.0001; PLSD *post hoc* test, Supplementary Figures 6A,B).

In the neurogenic niche of the dentate gyrus, new neurons are continuously generated from stem cells. Our data reveal that Snca is able to activate the proliferation of stem and type-2a progenitor cells (GFAP^+^Sox2^+^and Sox2^+^) in the defective Btg1 KO and mainly of type-2a progenitor cells in WT (*Btg1* WT Snca virus vs. *Btg1* WT empty virus, *p* < 0.0001, Figure 6C); thus, Snca behaves as a neurogenic activatory stimulus for stem cells.

Of note, very few neurogenic stimuli are able to reactivate stem cells (Ceccarelli et al., 2020), and this further suggests that Snca is potentially able to reconstitute the neural plasticity of the neurogenic niche reduced by aging.

Interestingly, our data showing a role of Snca as activator of dentate gyrus stem cells, are consistent with previous findings indicating that Snca is required to maintain stem cells of the SVZ in a cycling state (Perez-Villalba et al., 2018).

#### Control of Neuron Death by *Snca* and Rescue of Apoptosis by *Snca*-Virus in the Dentate Gyrus of the *Btg1* KO Model

One of the most evident features of the *Btg1* KO is the increased apoptosis of the dentate gyrus adult progenitor cells and neurons (Farioli-Vecchioli et al., 2012).

Snca is most known for its aggregates that form insoluble inclusions, detected in neurodegenerative diseases such as Parkinson’s disease (see for review, Ryskalin et al., 2018). These pathologies, however, are associated either with increased amounts of normal Snca, or with its mutated forms (Ryskalin et al., 2018).

However, the normal physiological function of Snca has not yet been fully elucidated. In fact, physiologically expressed Snca has been shown to exert a neuroprotective effect, as it inhibits apoptosis induced by several types of apoptotic stimuli, or to regulate the expression of proteins implicated in the apoptotic pathways (Sidhu et al., 2004). For instance, physiological concentrations of wild-type Snca reduce the activity and expression of the proapoptotic proteins p53 and Caspase-3 in the neuronal cell line TSM1, in the presence of apoptotic stimuli such as staurosporine, etoposide and ceramide (da Costa et al., 2000; Alves Da Costa et al., 2002). Moreover, physiological concentrations of Snca protect from apoptosis induced by serum withdrawal or H2O2, or oxidative stress, serum deprivation and excitotoxicity by glutamate, or also by rotenone, in hippocampal neurons and in neural cells such as TSM1, PC12, or SY-5Y (Lee et al., 2001; Hashimoto et al., 2002; Seo et al., 2002; Liu et al., 2006). The mechanism underlying this anti-apoptotic action seems to depend on the activation of the PI3/Akt pathway and on the down-regulation of Bcl2 (Seo et al., 2002).

Thus, we reasoned that the extremely low levels of Snca found in the *Btg1* KO mice may account for the observed increase of apoptosis. To test this possibility, we analyzed the number of dentate gyrus cells positive for activated Caspase-3 in the dentate gyrus of *Btg1* WT and KO mice, 5 days after injection of a lentivirus expressing Snca or empty. We observed that after injection with empty virus, the dentate gyrus of *Btg1* KO mice showed 76% increase of Caspase-3^+^ cells relative to *Btg1* WT; conversely, the injection of Snca virus in *Btg1* KO mice reduced the number of Caspase-3^+^ to control values (Btg1 KO empty virus vs. Btg1 WT empty virus, 76% increase, *p* = 0.040, *n* = 120 sections; Btg1 KO Snca virus vs. Btg1 WT empty virus, *p* = 0.84, *n* = 148 sections; Btg1 KO Snca virus vs. Btg1 KO empty virus, 44% decrease, *p* = 0.022, *n* = 127 sections; Mann-Whitney *U*-test, Figures 6D,E and Supplementary Figure 5B). The rescue by exogenous Snca supports the hypothesis that the increased apoptosis observed in *Btg1* KO dentate gyrus was a consequence of the down-regulation of Snca occurring in *Btg1* KO, and that Snca is endowed with neuroprotective functions in the dentate gyrus.

Furthermore, we wondered whether a possible mechanism for the extremely low levels of Snca observed in the *Btg1* KO dentate gyrus might depend on the ability of Btg1 to transactivate the *Snca* promoter.

**FIGURE 7:**
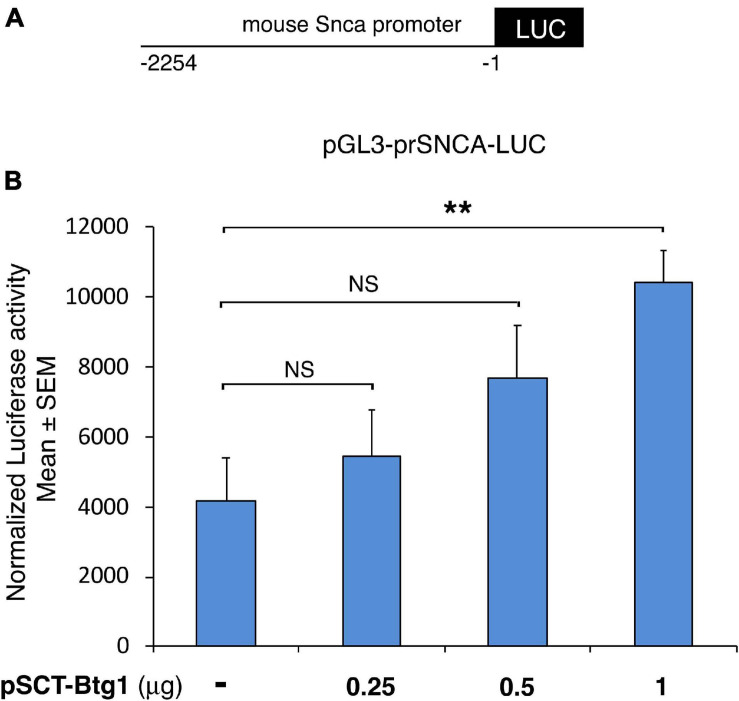
Mouse *alpha-synuclein* promoter activation by Btg1. *Alpha synuclein* promoter activity increased significantly in cells overexpressing Btg1 relative to the corresponding controls expressing endogenous levels of Btg1. **(A)** schematic representation of the alpha-synuclein promoter construct pGL3-prSNCA-LUC. −1 is referred to the translation initiation codon of the mouse *Snca* gene. **(B)** Promoter transactivation was analyzed after transient overexpression of pSCT empty vector (pSCT-empty) or of vector encoding for mouse Btg1 (pSCT-Btg1) in HEK293 cells. Luciferase activity normalized for Renilla luciferase is represented as mean ± SEM from three separate experiments. NS > 0.05, **p < 0.01 vs. the control without exogenous Btg1, Student’s *t*-test.

We checked whether the *Snca* promoter was activated by Btg1, by co-transfecting in HEK293T cells the pGL3-prSnca-LUC reporter with increasing amounts of an expression construct for Btg1 (pSCT-Btg1). We found that Btg1 is capable to significantly and dose-dependently activate the Snca promoter (pSCT-Btg1 1.0 μg, *p* = 0.003, Student’s *t*-test; Figure 7). Moreover, it is known that Snca inhibits p53 expression and transcriptional activity, in this way protecting from apoptosis (Alves Da Costa et al., 2002); conversely, p53 directly transactivates the *Snca* promoter (Duplan et al., 2016). These findings, as a whole, are consistent with the increased number of p53-expressing neural cells found in the *Btg1* KO dentate gyrus (Farioli-Vecchioli et al., 2012). Therefore, plausibly, Btg1 activates *Snca* promoter and expression, which, in turn, inhibits p53 expression, resulting in antiapoptotic activity; the opposite may occur if *Btg1* is deleted, suggesting that the increase of apoptosis present in Btg1 KO dentate gyrus could be ascribed to the absence of Snca.

#### Regulation of Neuronal Synaptic Plasticity (*Arc*, *Snca*) or of Dendritic Spine Morphogenesis (*Arc*)

It is interesting to note that amongst the genes analyzed (see Table 2), some are involved in the processes of neuronal plasticity. In particular, Snca plays, as mentioned above, a critical role in synaptic vesicle recycling, neurotransmitter synthesis and release and synaptic plasticity (Ghiglieri et al., 2018).

Indeed, the suppression of wild-type *Snca* from cultured hippocampal neurons blocks the long-term potentiation (LTP), while the introduction of Snca into the presynaptic neuron of a pair of cells connected by a single synapsis not only rescues the LTP block but induces a long-lasting enhancement of transmission; this indicates that Snca increases the transmitter release from the nerve terminal (Liu et al., 2004).

Also Arc, a cytoskeleton-associated protein, plays a key role in regulating synaptic plasticity, as it acts as a hub protein that binds to a network of proteins involved in glutamate receptor endocytosis, Notch signaling and dendritic spine growth and morphology, in this way signaling the synaptic strength through different molecular mechanisms (see for review Korb and Finkbeiner, 2011; Nikolaienko et al., 2018). It has been observed that overexpression of Arc in hippocampal cells increases spine density and the proportion of thin spines (Peebles et al., 2010). Although *Arc* knockout does not influence spine numbers (Plath et al., 2006), however, Arc is required for the elimination of synapses in the hippocampus (Wilkerson et al., 2014), and, more generally, Arc appears to be involved in the actin dynamics. Consistently with the fact that the morphological changes in dendritic spines impact on long-term plasticity (Yuste and Bonhoeffer, 2001), Arc is specifically required in the hippocampus for long-term potentiation and for the consolidation of long-term memory (Guzowski et al., 2000). Furthermore, Arc is activated very rapidly in response to several behavioral and learning paradigms (Ramírez-Amaya et al., 2005), including running (Clark et al., 2011), which all influence synaptic activity.

In fact, running enhances the plasticity of new neurons of the dentate gyrus by increasing spine density and dendritic complexity (Eadie et al., 2005; Stranahan et al., 2007), short-term synaptic plasticity and integration of spatial and contextual spatial information (Vivar et al., 2016), with corresponding changes in genes related to plasticity, growth factors and neurotransmitters in the dentate gyrus (Molteni et al., 2002; Lista and Sorrentino, 2010).

Since not only Snca but also Arc are significantly reduced in *Btg1* KO relative to *Btg1* WT (set B), and this deficit is fully reverted in *Btg1* KO by running, future studies may test the possibility of a change in the morphology of dendrites in *Btg1* KO mice, before and after running.

#### Long Term-Memory and Learning (*Arc*, *Npas4, Egr1*) - Regulation of GABAergic Synaptic Transmission (*Npas4*) - Short-Term Memory (*Npas4*)

The *Btg1* KO shows an impaired pattern separation, and running rescues that deficit (Farioli-Vecchioli et al., 2014). Consistent with this, the GO database analysis reveals biological processes related to memory formation (with genes *Arc*, *Npas4*, and *Egr1*).

In addition to *Arc*, *Npas4* is another key activity-dependent immediate early gene downregulated in Set B and up-regulated in Set A (Figure 3). Npas4 is a transcription factor that regulates neural circuits plasticity, is expressed only in neurons and is required for long-term memory formation in hippocampus and amygdala (for review see Sun and Lin, 2016).

In fact, deletion of *Npas4* causes a defect of contextual memory, detectable as impairment of contextual fear conditioning (Ramamoorthi et al., 2011). It is worth noting that *Btg1* KO mice show a significant reduction of Npas4 which may thus be responsible for the observed loss of contextual memory (Farioli-Vecchioli et al., 2012). Npas4 appears to positively regulate the inhibitory synapse of GABAergic synaptic inputs in the dentate gyrus neurons during learning with a mechanism involving its downstream gene *Bdnf* (Lin et al., 2008; Bloodgood et al., 2013). In fact, reducing Npas4 expression in cultured hippocampal pyramidal neurons leads to a reduced number of inhibitory synapses, while Npas4 overexpression increases their number (Lin et al., 2008).

Notably, Npas4 expression is induced by different proneurogenic stimuli, such as enriched environment (Bloodgood et al., 2013), the antidepressant fluoxetine (Maya-Vetencourt et al., 2012) or cerebral ischemia (Shamloo et al., 2006). Moreover, treatments leading to long-term contextual memory are able to induce Npas4, while the footshock alone is not sufficient, although it is an effective inducer of Fos and Arc (Ramamoorthi et al., 2011).

Consistently with the above functional findings on Npas4, we observe that the neurogenic induction by running in *Btg1* KO dentate gyrus rescues the defect of contextual memory (Farioli-Vecchioli et al., 2014) and, in parallel, strongly up-regulates the expression of Npas4 (Figures 3, 5). This suggests a correlation between the two events observed.

Concerning the underlying mechanisms, Npas4 is able to activate arrays of genes by binding directly to their promoter: the acute deletion of Npas4 greatly reduces several activity-induced genes, included the majority of IEGs, such as *Fos* and *Arc* (Ramamoorthi et al., 2011), a fact that may account for the significant decrease of mRNA of *Fos* and *Arc* observed in *Btg1* KO (set B). Moreover, constitutive deletion of *Npas4* impairs also short-term memory (Ramamoorthi et al., 2011). All this suggests that Npas4 acts upstream of several activity-regulated genes, thus effectively regulating synaptic activity.

**FIGURE 8:**
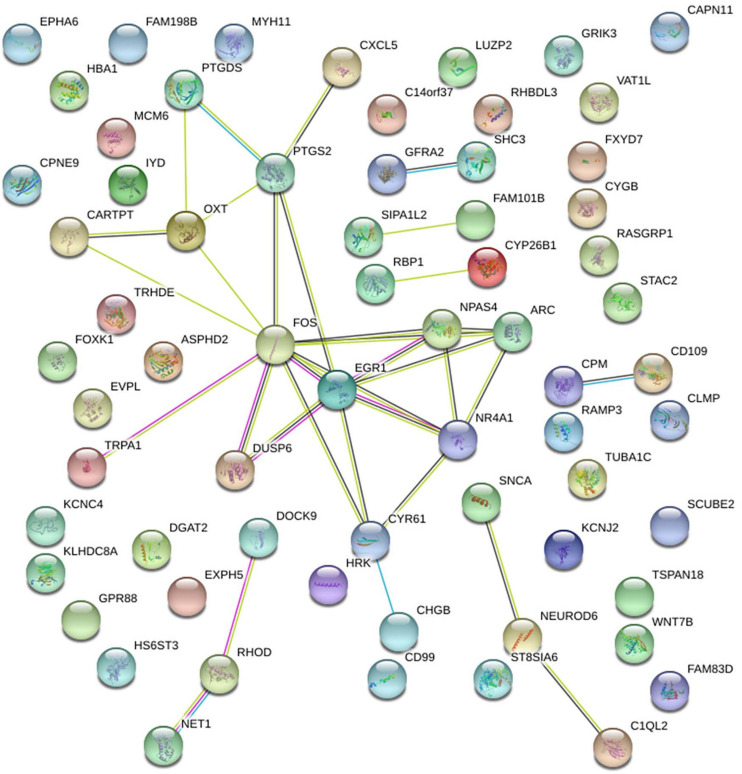
Protein-Protein interaction analysis by the software STRING of the genes either down-regulated or up-regulated in set B by *Btg1* knockout and counter-regulated by running, in set A. A network of interacting proteins is configured, with Fos, Egr1, Npas4 and Arc representing hubs of this network.

Interestingly, we observed by protein-protein interaction analysis of the genes counter-regulated by running in set A (vs. set B), using the software STRING, that Arc, Fos, Npas4 and Egr1 proteins are central hubs of a network of interacting proteins (Figure 8). Interestingly, Egr1 shares a critical role in this gene network being activator of the transcription of the GABAA receptor subunits, which is a key regulator of hippocampal neurogenesis (Mo et al., 2015).

#### Aging (*Fos*, *Snca*, *Ptgs2*)

The expression of Snca in the rodent brain is highest in the hippocampus and cortex (Malatynska et al., 2006), and reaches a peak early postnatally, while during aging it decreases, more markedly at mRNA level than protein (Petersen et al., 1999; Mak et al., 2009). We have also observed a decrease of Snca mRNA expression in the dentate gyrus of 15-month-old mice, relative to 2-month-old mice (Supplementary Figure 7). A decrease of Snca is observed also during aging in humans (Daniele et al., 2018). Furthermore, in the autoptic Baltimore study of aging, very few cases were found where Snca was associated to brain lesions in aged healthy control subjects, as opposed to cases of neurodegenerative diseases (Mikolaenko et al., 2005). This suggests that the Snca pathology in neurodegenerative disease does not overlap with the physiological decrease of Snca in aging (Daniele et al., 2018). Based on our observations on Snca overexpression in the Btg1 model, we hypothesize that during physiological aging, the downregulation of Snca could be one factor responsible for the reduced neurogenesis observed. It is worth noting that 12 days of running led to an increased expression of Snca in the dentate gyrus of middle-aged mice (Supplementary Figure 7).

Moreover, a marked decrease of Ptgs2 was observed in the aging dentate gyrus and CA2-CA3 hippocampal subfields (Jung et al., 2017). These findings would be consistent with our data of reduced expression levels of *Snca* and *Ptgs2* genes in *Btg1* KO dentate gyrus, which we assume to be a model of neural aging; these decreases are counter-regulated by running (Figures 3, 5). In contrast, no change of expression in the hippocampus during aging was observed for Fos (Desjardins et al., 1997). However, *Fos* has been associated to aging, as in aged hippocampi the AP-1 (Activator protein-1) complex, of which Fos is part, displays a reduced transcriptional response to oxidative stress (Tong et al., 2002).

Moreover, the expression of the N-myc downstream-regulated gene 2 (*Ndrg2*), involved in proliferation, differentiation and apoptosis, and of *Drosophila melanogaster* rhomboid protease homolog *Rhbdl3* gene, have been observed to be increased during aging (Kumar et al., 2013; Rong et al., 2017), and they are highly induced as well by *Btg1* deletion (Set B), although only *Rhbdl3* mRNA is counter-regulated by running in Set A (Set B: *Ndrg2* and *Rhbdl3 p* < 0.0001; Set A: *Ndrg2 p* = 0.87 and *Rhbdl3 p* = 0.038; Figure 4). This further supports our idea that *Btg1* KO is a model of neural aging.

## Conclusion

Thus, several pieces of correlative evidence link the increased apoptosis and decreased neurogenesis of this *Btg1* KO model of accelerated aging to the strong decrease of *Snca*, *Arc*, and *Npas4* transcripts expression observed here. In parallel we demonstrate here that running is able to rescue the defective expression of these genes in our model of premature aging. It is known that running is able to rescue neurogenic functional deficits in young as well as aged mice. In fact, running increases cell survival by increasing BDNF levels and the phosphorylation of Akt, a key gene for cell survival (Chen and Russo-Neustadt, 2009). Moreover, running enhances the short-term plasticity by increasing the input from the entorhinal cortex, and consequently facilitating the contextual and spatial information afferent to the dentate gyrus (Vivar et al., 2016). As a result, running induces a rapid increase of proliferation of neural progenitor cells (Patten et al., 2013). Notably, however, running is not able to activate stem cells in WT mouse models (see for review Ceccarelli et al., 2020); we show here that Snca overexpression activates stem cells in *Btg1* KO model, being able to rescue the defective proliferation of stem cells, and boosts the proliferation of progenitor cells in WT mice. Therefore, we demonstrated that Snca represents an effector for stem and progenitor cell activation, and the rescue of *Snca* defective expression in *Btg1* KO dentate gyrus can be viewed as a neurogenic inducer against neural aging.

We think that in physiological conditions the balance between Snca and Btg1 expression can be responsible for the maintenance of stem cell self-renewal, and that *Snca* modulation may therefore play a central role as a regulator of neurogenesis in aged subjects.

Finally, the Snca overexpression rescues the excess of apoptosis seen in the *Btg1* KO mice, thus indicating that the whole process of neurogenesis in the dentate gyrus is activated and supported by Snca.

## Data Availability Statement

The RNA sequencing data presented in the study are deposited in the Gene Expression Omnibus (GEO) repository with accession number GSE179081.

## Ethics Statement

The animal study was reviewed and approved by the Italian Ministry of Health (authorization 442-2016-PR).

## Author Contributions

FT had the conceptual idea, analyzed the data, and wrote the manuscript. LM contributed to the conceptual idea and experimental design, performed the experimental work, and edited the manuscript. TC and NA performed the data analysis and contributed to writing and editing the text. GG, RC, RS, MC, and GD’A contributed to the experimental work. MC and GD’A gave suggestions and refined the manuscript. All authors contributed to the article and approved the submitted version.

## Conflict of Interest

The authors declare that the research was conducted in the absence of any commercial or financial relationships that could be construed as a potential conflict of interest.

## Publisher’s Note

All claims expressed in this article are solely those of the authors and do not necessarily represent those of their affiliated organizations, or those of the publisher, the editors and the reviewers. Any product that may be evaluated in this article, or claim that may be made by its manufacturer, is not guaranteed or endorsed by the publisher.

## Footnotes

1
https://igatechnology.com

2
https://www.ncbi.nlm.nih.gov/geo/

3
http://current.geneontology.org/annotations/

4
http://www.brainstars.org/

## Supplementary Material

Supplementary Figure 1 Gene Ontology enrichment of the 52 DE genes of Set C common to the Running set of Grégoire et al. (2018). An enrichment analysis for Gene Ontology Biological Processes was performed by implementing a Fisher’s exact test, using a subset of 52 genes, listed in Table 1. Their expression is up- or down-regulated by running in wild-type Btg1 dentate gyrus (Set C), and is concordantly up- or down-regulated by running as described in a previous analysis by Grégoire et al. (2018). Data settings are available in Table 1. GO term, category and description columns contain the identifier, the sub-ontology the term belongs to and the name of the GO process; number of DE genes are the number of genes in our list common to both set C and to the running Set by Grégoire et al. (2018), and mapping to the GO process; size list indicates the total number of genes in the indicated GO process; *p*-Value is the enrichment probability calculated using the Fisher’s exact test. All results shown have been considered valid for a *p*-Value < 0.05.

Supplementary Figure 2 Worksheet “Pairwise comparison of DE:” differentially expressed genes with *p*-Value < 0.05 of the Set B which are counter-regulated by running in other Sets. In each column are indicated the genes which are down- (B dw) or up-regulated (B up) in set B (*Btg1* knockout control vs. *Btg1* wild-type control) vs. all other sets as indicated. The chief pairwise comparisons show 42 genes down-regulated or up-regulated in Set B and correspondingly counter-regulated in Set A (A up B dw, and A dw B up, respectively). The genes were identified between data Sets A, B, C, D by performing pairwise comparisons. In worksheet “DE Genes Sets A-B-C-D *p* < 0.05” are listed all the differentially expressed genes with *p*-Value < 0.05 of each different Sets.

Supplementary Figure 3 Full list of GO processes enriched in DE genes down-regulated in Set B and up-regulated in Set A with *p* < 0.05 (representative GO processes are indicated in Table 2). The enrichment *p*-Value for each functional class has been calculated using the Fisher’s Exact test. GO term, category and description columns contain the identifier, the sub-ontology the term belongs to and the name of the GO process; number of DE genes is referred to the genes down-regulated in Set B and correspondingly up-regulated in Set A, mapping to the specific GO process; Size list is the total number of genes belonging to the specific GO process; *p*-Value is the enrichment probability calculated using the Fisher’s exact test.

Supplementary Figure 4 Full list of GO processes enriched in DE genes up-regulated in Set B and down-regulated in Set A with *p* < 0.05 (representative GO processes are indicated in Table 2). The enrichment *p*-Value for each functional class has been calculated using the Fisher’s Exact test. GO term, category and description columns contain the identifier, the sub-ontology the term belongs to and the name of the GO process; number of DE genes is referred to the genes down-regulated in Set A and correspondingly up-regulated in Set B, mapping to the specific GO process; Size list is the total number of genes belonging to a specific GO process; *p*-Value is the enrichment probability calculated using the Fisher’s exact test (*p*-Value).

Supplementary Figure 5 **(A)** Representative confocal images (20 × magnification) of coronal sections of the dentate gyrus of *Btg1* wild-type mice, relative to the experiment shown in Figure 6B for *Btg1* knockout mice, labeled with Ki67, Sox2 and GFP (in red, blue and green, respectively), 5 days after infection with either pCCL-Snca or pCCL-empty lentiviruses. The white arrows indicate triple-labeled cells for GFP, Ki67 and Sox2; white arrowheads indicate cells labeled for Ki67. Scale bars, 100 μm. **(B)** Representative images by confocal microscopy (40 × magnification) of apoptotic cells in the dentate gyrus of *Btg1* wild-type mice, relative to the experiment shown in Figure 6D for *Btg1* knockout mice, labeled with activated Caspase-3 (in red) and GFP (in green), 5 days after infection with either GFP-Snca or GFP-empty lentiviruses. Nuclei are counterstained with Hoechst 33258 (cyan). The white arrow indicates a cell double positive for GFP and activated Caspase-3 and the white arrowhead indicates a cell positive for Caspase-3. Scale bars, 50 μm.

Supplementary Figure 6 Rescue of the defective alpha-synuclein expression in *Btg1* knockout gyrus through virus-mediated transfer reactivates Ki67^+^ GFAP^+^ Sox2^+^ radial-glia like stem cell proliferation. **(A)** Representative confocal images (40 × magnification) of a coronal section of dentate gyrus quadruple-labeled with Ki67, GFAP Sox2, and GFP (in red, gray, blue, and green, respectively), with 3D reconstruction and orthogonal projection, showing radial-glia like cells. The dentate gyrus section shown belongs to the *Btg1* knockout mouse 5 days after infection with pCCL-Snca lentivirus. The orthogonal projection corresponds to the white box area, with 1.9 x digital magnification, and marks a radial-glia like quadruple labeled cell. Scale bars, 50 μm. **(B)** Quantification of the absolute number of proliferating dentate gyrus radial glia-like stem cells (type-1; Ki67^+^ GFAP^+^ Sox2^+^). We observe a significant decrease of stem cells in Btg1 KO dentate gyri infected with empty virus, relative to Btg1 WT, which is reversed above control levels in KO mice infected with pCCL-Snca virus. Analysis of simple effects: ^∗^*p* < 0.05; ^∗∗^*p* < 0.01; and ^****^*p* < 0.0001, or NS *p* > 0.05, two-way ANOVA, Fisher PLSD *post hoc* test. Cell numbers in the dentate gyrus are means ± SEM of the analysis of three animals per group. **(C)** The images shown are confocal images (20× magnification) of immunohistochemistry negative controls, where the primary antibodies were omitted (i.e., Ki67, Sox2, and GFAP) while the secondary antibodies were applied, as indicated (DAR-TRITC, donkey anti-rabbit TRITC; DAG-AF647, donkey anti-goat Alexa Fluor 647; DAM-DL405, donkey anti-mouse DyLight 405). The green signal represents cells infected with the pCCL lentivirus and expressing GFP. The white dotted line shows the dentate gyrus boundaries. Scale bar, 100 μm.

Supplementary Figure 7 Real-time PCR analysis in the dentate gyrus, isolated from 2-month-old or from 15-month-old wild-type mice, submitted to physical exercise or sedentary. Shown are the mean mRNA expression fold increases ± SEM from three independent experiments. One-way ANOVA, treatment and age effect: Snca *F*(3,10) *p* = 0.0018; Fisher PLSD *post hoc* test: 15-month-old CTL vs. 2-month-old CTL *p* = 0.041; 15-month-old RUN vs. 15-month-old CTL *p* = 0.0002; 2-month-old RUN vs. 2-month-old CTL *p* = 0.56. TBP was used to normalize data. ^∗^*p* < 0.05, ^∗∗∗^*p* < 0.001, or NS *p* > 0.05, one-way ANOVA, Fisher PLSD *post hoc* test.

Supplementary Table 1 Specific RT-PCR primers used, deduced from published murine cDNA sequences.

## Abbreviations

ANOVA: analysis of variance
Arc: activity regulated cytoskeleton associated protein
Bdnf: brain-derived neurotrophic factor
Btg1: B-cell translocation 1 gene
DE: differentially expressed
DG: dentate gyrus
Egr-1: early growth response protein 1
FPKM: fragments per Kilobase Million
GFP: green fluorescent protein
GO: Gene Ontology
Igf-2: insuline-like growth factor 2
KO: knockout
Npas4: neuronal PAS domain protein 4
PFA: paraformaldehyde
PBS: phosphate buffered saline
PLSD: protected least significant difference
*Ptgs2*: Prostaglandin-Endoperoxide Synthase 2
SEM: standard error of the mean
*Snca*: alpha-synuclein
Sox2: sex determining region Y-box 2
SVZ: subventricular zone
WT: wild-type.
