# Supplementary figures and images for: Transcriptome Analysis in a Mouse Model of Premature Aging of Dentate Gyrus: Rescue of Alpha-Synuclein Deficit by Virus-Driven Expression or by Running Restores the Defective Neurogenesis
Source: Front Cell Dev Biol. 2021 Aug 17;9:696684. doi: 10.3389/fcell.2021.696684 (PMC8415876; doi:10.3389/fcell.2021.696684)

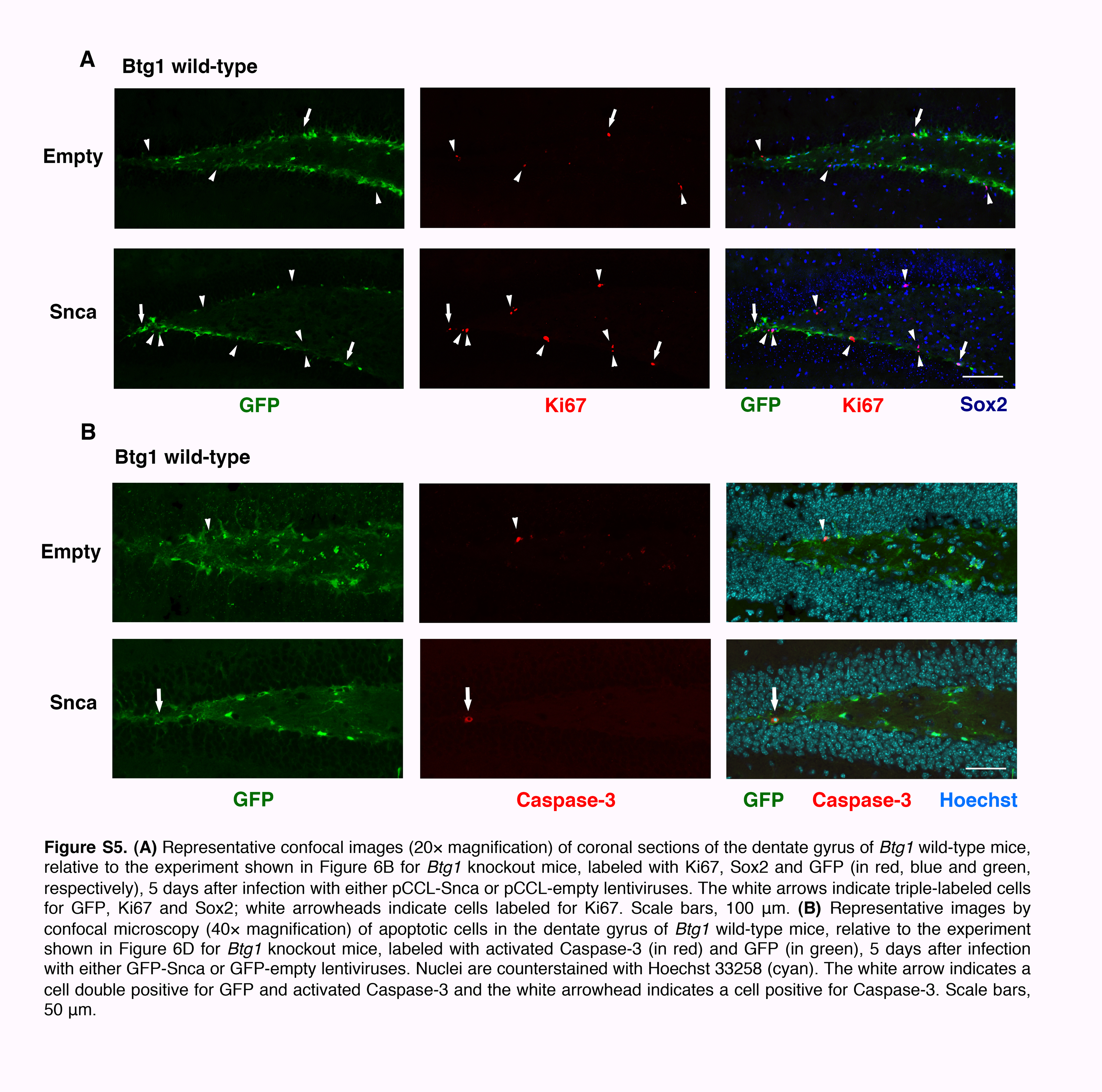

Supplement: Supplementary Figure 5 — (A) Representative confocal images (20 × magnification) of coronal sections of the dentate gyrus of Btg1 wild-type mice, relative to the experiment shown in Figure 6B for Btg1 knockout mice, labeled with Ki67, Sox2 and GFP (in red, blue and green, respectively), 5 days after infection with either pCCL-Snca or pCCL-empty lentiviruses. The white arrows indicate triple-labeled cells for GFP, Ki67 and Sox2; white arrowheads indicate cells labeled for Ki67. Scale bars, 100 μm. (B) Representative images by confocal microscopy (40 × magnification) of apoptotic cells in the dentate gyrus of Btg1 wild-type mice, relative to the experiment shown in Figure 6D for Btg1 knockout mice, labeled with activated Caspase-3 (in red) and GFP (in green), 5 days after infection with either GFP-Snca or GFP-empty lentiviruses. Nuclei are counterstained with Hoechst 33258 (cyan). The white arrow indicates a cell double positive for GFP and activated Caspase-3 and the white arrowhead indicates a cell positive for Caspase-3. Scale bars, 50 μm. [file Image_1.TIF]

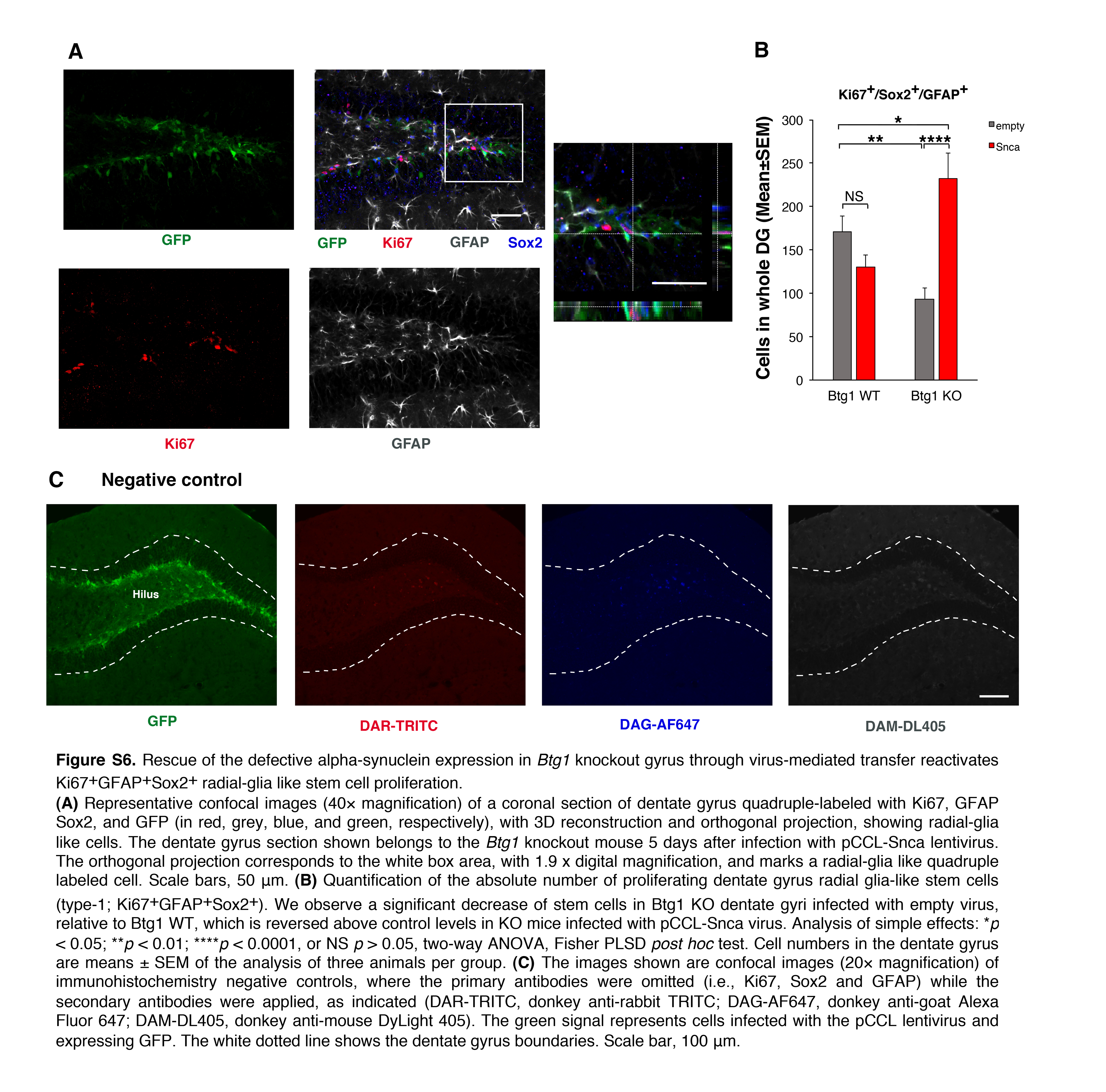

Supplement: Supplementary Figure 6 — Rescue of the defective alpha-synuclein expression in Btg1 knockout gyrus through virus-mediated transfer reactivates Ki67+ GFAP+ Sox2+ radial-glia like stem cell proliferation. (A) Representative confocal images (40 × magnification) of a coronal section of dentate gyrus quadruple-labeled with Ki67, GFAP Sox2, and GFP (in red, gray, blue, and green, respectively), with 3D reconstruction and orthogonal projection, showing radial-glia like cells. The dentate gyrus section shown belongs to the Btg1 knockout mouse 5 days after infection with pCCL-Snca lentivirus. The orthogonal projection corresponds to the white box area, with 1.9 x digital magnification, and marks a radial-glia like quadruple labeled cell. Scale bars, 50 μm. (B) Quantification of the absolute number of proliferating dentate gyrus radial glia-like stem cells (type-1; Ki67+ GFAP+ Sox2+). We observe a significant decrease of stem cells in Btg1 KO dentate gyri infected with empty virus, relative to Btg1 WT, which is reversed above control levels in KO mice infected with pCCL-Snca virus. Analysis of simple effects: ∗p < 0.05; ∗∗p < 0.01; and ****p < 0.0001, or NS p > 0.05, two-way ANOVA, Fisher PLSD post hoc test. Cell numbers in the dentate gyrus are means ± SEM of the analysis of three animals per group. (C) The images shown are confocal images (20× magnification) of immunohistochemistry negative controls, where the primary antibodies were omitted (i.e., Ki67, Sox2, and GFAP) while the secondary antibodies were applied, as indicated (DAR-TRITC, donkey anti-rabbit TRITC; DAG-AF647, donkey anti-goat Alexa Fluor 647; DAM-DL405, donkey anti-mouse DyLight 405). The green signal represents cells infected with the pCCL lentivirus and expressing GFP. The white dotted line shows the dentate gyrus boundaries. Scale bar, 100 μm. [file Image_2.TIF]

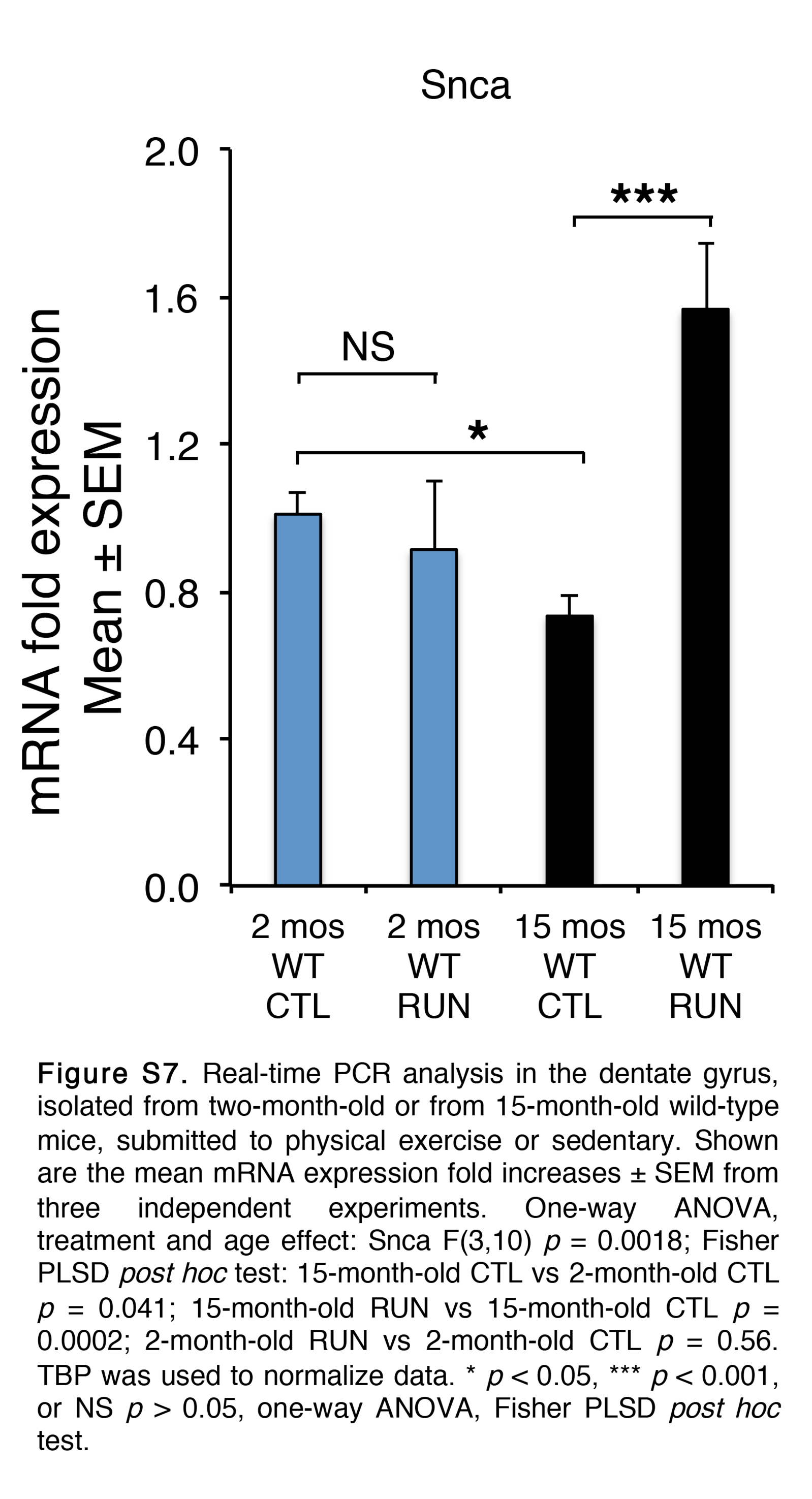

Supplement: Supplementary Figure 7 — Real-time PCR analysis in the dentate gyrus, isolated from 2-month-old or from 15-month-old wild-type mice, submitted to physical exercise or sedentary. Shown are the mean mRNA expression fold increases ± SEM from three independent experiments. One-way ANOVA, treatment and age effect: Snca F(3,10) p = 0.0018; Fisher PLSD post hoc test: 15-month-old CTL vs. 2-month-old CTL p = 0.041; 15-month-old RUN vs. 15-month-old CTL p = 0.0002; 2-month-old RUN vs. 2-month-old CTL p = 0.56. TBP was used to normalize data. ∗p < 0.05, ∗∗∗p < 0.001, or NS p > 0.05, one-way ANOVA, Fisher PLSD post hoc test. [file Image_3.TIF]
