# Supplementary material for: Transcriptome Analysis in a Mouse Model of Premature Aging of Dentate Gyrus: Rescue of Alpha-Synuclein Deficit by Virus-Driven Expression or by Running Restores the Defective Neurogenesis
Source: Front Cell Dev Biol. 2021 Aug 17;9:696684. doi: 10.3389/fcell.2021.696684 (PMC8415876; doi:10.3389/fcell.2021.696684)
Supplement: Supplementary Table 1 — Specific RT-PCR primers used, deduced from published murine cDNA sequences. [file Table_1.DOCX]

Table S1. Specific RT-PCR primers used, deduced from published murine cDNA sequences.

| Gene | Primer Sequence (5’-3’) |
| --- | --- |
| Arc | F: TGAGCTGAAGCCACAAATGC  R: GGTATGAATCACTGCTGGGGG |
| BDNF | F: GGACTCTGGAGAGCGTGAATGG  R: AGTGTCAGCCAGTGATGTCGTC |
| Egr1 | F: CACCTGACCACAGAGTCCTTT  R: TAACTCGTCTCCACCATCGC |
| Fos | F: TACTACCATTCCCCAGCCGA  R: GCGCAAAAGTCCTGTGTGTT |
| IGF2 | F: GGAGGGGAGCTTGTTGACAC  R: AGGCCTGCTGAAGTAGAAGC |
| Npas4 | F: GTATGGACTGCTACACCCCG  R: CTTTCAGCCAACAGGCGGTA |
| Ptgs2 | F: TGAGTACCGCAAACGCTTCT  R: CAGCCATTTCCTTCTCTCCTGT |
| Snca | F: CTGCCCTTGCCTCTTTCATTG  R: TTTTTGCTCCACACGGCTCC |
| TBP | F: CCAATGACTCCTATGACCCCTA  R: CAGCCAAGATTCACGGTAGAT |
| F, forward; R, reverse. |  |
